# A Low-Loss Hybrid Acoustic Bandpass Filter with Continuously Tunable Fractional Bandwidth

**DOI:** 10.3390/mi17091101

**Published:** 2026-09-21

**Authors:** Xianli Tang, Yonghao Jia, Yuandong Gu

**Affiliations:** 1 School of Microelectronics, Shanghai University, Shanghai 200444, China; tangxl1229@gmail.com; 2 State Key Laboratory of Millimeter Waves and School of Information Science and Engineering, Southeast University, Nanjing 210096, China

**Keywords:** acoustic wave resonator, fractional bandwidth, hybrid filter, reconfigurable bandpass filter, varactor-tuned

## Abstract

This research proposes a low-loss hybrid acoustic bandpass filter with continuously tunable fractional bandwidth. A reconfigurable acoustic-wave-lumped-element resonator (AWLR) module with a varactor diode is studied. Based on the reconfigurable AWLR modules, two ladder-type tunable filters, including a single-series/single-shunt configuration (Type I) and a single-series/two-shunt configuration (Type II), are designed. Their analytical expressions are derived to directly predict their electrical characteristics. Their calculated and simulated insertion loss and 3 dB fractional bandwidth show good agreement, which validates the correctness of the theoretical analysis. The proposed two configurations of the reconfigurable AWLR-based filters are fabricated and measured. The Type I filter achieves a 3 dB fractional bandwidth tuning range from 1.16*k_t_*^2^ to 1.76*k_t_*^2^ with a minimum insertion loss of 1.2 dB and a maximum of 2.2 dB. The Type II filter provides a wider tuning range from 0.96*k_t_*^2^ to 1.85*k_t_*^2^ with a minimum insertion loss of 1.7 dB and a maximum of 2.7 dB. The results indicate that the proposed filter offers a favorable balance between bandwidth tunability and insertion loss.

## 1. Introduction

The evolution of 5G and 6G communications is driving an ever-increasing demand for multifunctional RF front-ends [1]. The communication systems are required to support diverse applications, including distinct frequency bands, operational modes, and bandwidth requirements [2,3]. The multi-standard and multi-band operation introduces significant challenges for RF front-end design. Conventionally, each frequency band needs a dedicated fixed-frequency filter. Thus, multiple frequency bands require a large number of filters, switches, and matching networks, which can increase the complexity of communication systems and hinder the system miniaturization [4]. In response to these challenges, bandpass filters (BPFs) with electronically reconfigurable transfer functions offer an effective path to enhance system flexibility and reduce physical size [5,6]. Unlike conventional filter modules, which require numerous fixed-frequency filters and switching networks, reconfigurable filters provide advantages in terms of reduced size, lower system complexity, and improved spectral efficiency [7].

Reconfigurable filters are typically implemented using tunable elements such as PIN diodes or varactor diodes, which enable dynamic adjustment of the frequency response [8]. A wide variety of reconfigurable filters have been reported, such as microstrip-based tunable filters, planar transmission-line reconfigurable filters, and coaxial/waveguide-based tunable filters [9,10]. The majority of these tunable filters are large in size or are based on low-quality factor (Q) resonators. The value of Q is typically around 50–150 for microstrip-type configurations [9]. The in-band insertion loss of these filters has been reported to range from 3 dB to 10 dB [8,11,12]. In another approach, RF filters based on surface acoustic wave (SAW) or bulk acoustic wave (BAW) resonators can offer Q factors in the thousands and a chip-sized dimension [13,14], benefiting from MEMS fabrication technologies that enable miniaturization and integration, as demonstrated in various MEMS devices reported in [15,16,17]. A Sc_0.2_Al_0.8_N BAW filter with Q factors exceeding 1000 and insertion loss below 2 dB is reported [18]. A LiNbO_3_ SAW resonator fabricated at the wafer level, which achieves a 3 GHz operation frequency, is studied [19]. Despite these achievements, conventional acoustic wave (AW) filters employing ladder, lattice, or cascaded topologies are constrained by two limitations. First, their fractional bandwidth (FBW) is intrinsically limited by the electromechanical coupling coefficient (*k_t_*^2^) of the piezoelectric substrate. Their 3 dB FBW usually falls within 0.4*k_t_*^2^–0.8*k_t_*^2^ [20]. Second, they have fixed passband frequencies and lack the reconfigurability required by modern adaptive transceivers [21].

To overcome the FBW limitations of the AW filter, a hybrid filter combining acoustic wave resonators (AWRs) and lumped-element components has emerged as a promising solution method [22,23]. In the acoustic-wave-lumped-element resonator (AWLR) configurations, inductors or capacitors are combined with AWRs to decouple the resonance frequency from the anti-resonance frequency of the AWR [22]. It can enable FBW to exceed the conventional 0.4*k_t_*^2^–0.8*k_t_*^2^ limit while preserving the inherent high-Q characteristics of acoustic devices [22]. In our recent work, the FBW enhancement of AWLR-based filters is investigated [24,25]. The measured FBWs are 1.63*k_t_*^2^, 1.94*k_t_*^2^, and 1.86*k_t_*^2^ [24,25]. However, all of the aforementioned AWLR-based filters remain fixed-frequency and cannot offer the tunability once fabricated. The reconfigurable capability is increasingly critical for multi-standard communication systems.

To achieve reconfigurability, the various reconfigurable functionalities based on the AWLR-based filters have been studied [26,27,28,29]. Multiband AWLR-based bandpass-to-bandstop (BP-to-BS) filters have been demonstrated [26]. An RF switch is employed to change the source-to-load coupling path to make the filter switch between bandpass and bandstop responses [26]. All-pass-to-bandstop reconfigurable AWLR filters have been reported [27]. It employs a varactor-tuned phase shifter to achieve a continuous transition between all-pass and bandstop responses [27]. Single-to-multi-band reconfigurable AWLR BPFs with PIN diodes that achieve multiple parallel bandpass channels have been presented in [28]. In addition, hybrid SAW-resonator/lumped-element dual-band bandstop filters with continuously tunable stopband bandwidths have been reported in [29]. It is noted that those works demonstrate various reconfigurable functions, including mode switching, bandpass selection, and stopband tunability.

Unlike the aforementioned works, the AWLR-based bandpass filters with continuously tunable bandwidth have been reported in [30,31]. A four-stage quasi-elliptic AWLR BPF with varactor-based continuous FBW tuning is demonstrated, achieving a tunable FBW ranging from 0.57*k_t_*^2^ to 1.32*k_t_*^2^ [30]. The configurable AWLR-based BPF is designed using multibranch topologies with multiple tunable low-Q lumped-element resonators and impedance inverters [6]. It offers continuous center frequency and FBW tuning [6]. More recently, the BP-to-BS reconfigurable filters with switchless tuning of both center frequency and bandwidth have been reported in [31]. These great works highlight the potential of AWLR-based filters for electronic tunability of the bandpass bandwidth. However, they rely on complex multibranch topologies with multiple tunable resonators and impedance inverters, which inevitably increase circuit complexity and insertion loss [30,31]. The maximum insertion loss is 7.3 dB [6]. A balance between reconfigurability and insertion loss is essential in the tunable AWLR-based filter.

In this paper, to address the trade-off between bandwidth tunability and insertion loss, a low-loss hybrid acoustic filter with continuously tunable fractional bandwidth is proposed. A reconfigurable AWLR module with a varactor diode is employed to design a ladder-type tunable filter. Two configurations, named Type I (single-series/single-shunt configuration) and Type II (single-series/two-shunt configuration), are studied. A theoretical analysis based on the two designed configurations is presented, which can directly predict the electrical characteristics of the reconfigurable hybrid filter. The calculated insertion loss and 3 dB fractional bandwidth are validated by simulations, showing good agreement. The two configurations are fabricated and measured. The Type I filter achieves a fractional bandwidth tuning range from 1.16*k_t_*^2^ to 1.76*k_t_*^2^ with a minimum insertion loss of 1.2 dB and a maximum of 2.2 dB. The Type II filter provides a wider tuning range from 0.96*k_t_*^2^ to 1.85*k_t_*^2^ with a minimum insertion loss of 1.7 dB and a maximum of 2.7 dB. The results indicate that the proposed filter offers a favorable balance between bandwidth tunability and insertion loss. The rest of the article is organized as follows. Section 2 presents the theoretical analysis and simulation results of the proposed reconfigurable hybrid filters. In Section 3, the proposed tunable filters are verified against the measurement data, and a comparison with reported works is also presented. In the end, Section 4 concludes the paper.

## 2. Theoretical Foundations

### 2.1. Reconfigurable AWLR Module

**Figure 1:**
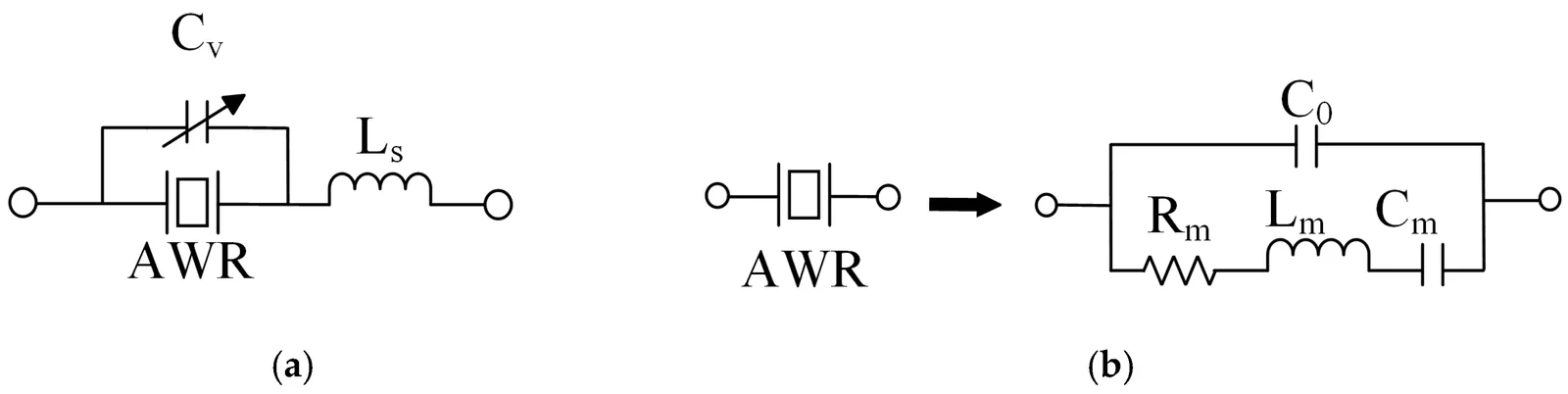
(**a**) Reconfigurable AWLR module; (**b**) Butterworth–Van Dyke (BVD) circuit model.

The proposed reconfigurable AWLR module is presented in Figure 1a. It consists of an AWR, a series inductor *L_s_*, and a shunt tunable capacitor *C_v_*. The AWR can be modeled by the Butterworth–Van Dyke (BVD) model, as shown in Figure 1b. The series inductor *L_s_* is employed to change the resonant frequency *f_s_* of the AWR. The shunt tunable capacitor *C_v_* is used primarily to adjust the anti-resonant frequency *f_p_* of the AWR. The inductor *L_s_* and capacitor *C_v_* are combined with AWRs to decouple the resonance and the anti-resonance of the AWR. It can enable the hybrid filter to achieve a 3 dB FBW that exceeds the conventional 0.4*k_t_*^2^–0.8*k_t_*^2^ limit of AW filters. By tuning the value of *C_v_*, the frequency response of the reconfigurable AWLR module can be continuously tuned.

**Figure 2:**
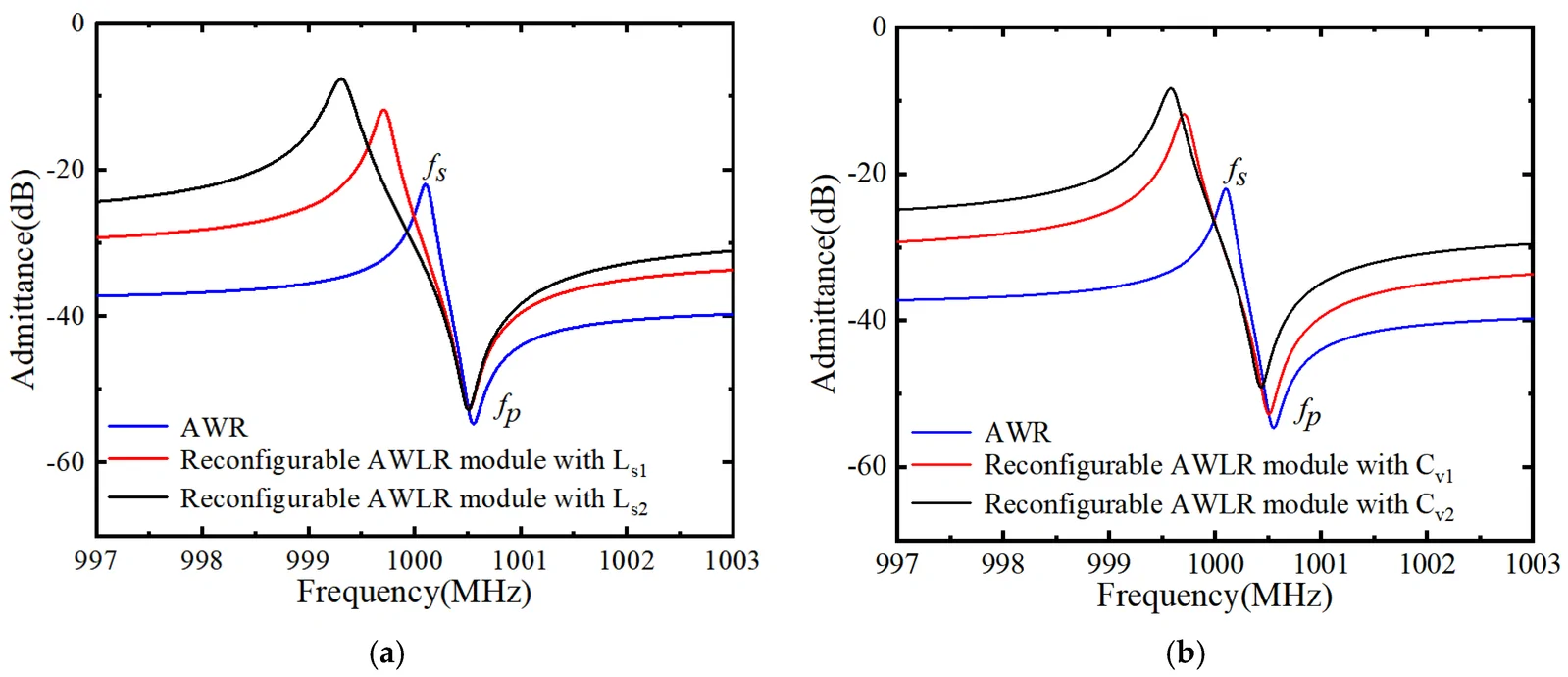
Admittance response of the reconfigurable AWLR module: (**a**) Admittance response with different values of *L_s_*; (**b**) admittance response with different values of *C_v_*. (The values of *L_m_*, *C_m_*, *R_m_*, and *C*_0_ are 15.265 μH, 1.659 fF, 12.8 Ω, and1.93 pF, respectively).

With fixed values of the tunable capacitor *C_v_*, the admittance response of the configurable AWLR module with different values of the series inductor *L_s_* is plotted in Figure 2a. The blue line represents the admittance response of the AWR. The simulated results indicate that the series inductor *L_s_* can be used to flexibly adjust the resonant frequency *f_s_* of the AWR. With fixed values of the series inductor *L_s_*, the admittance response of the configurable AWLR module with different values of *C_v_* is plotted in Figure 2b. The simulated results show that the tunable capacitor *C_v_* can not only change the anti-resonant frequency *f_p_* but can also influence the resonant frequency *f_s_* of the AWR. Since the employed *L_s_* is a fixed value for designing the reconfigurable AWLR-based filter, the tunable FBW is realized by the tunable capacitor *C_v_* of the configurable AWLR module.

**Figure 3:**
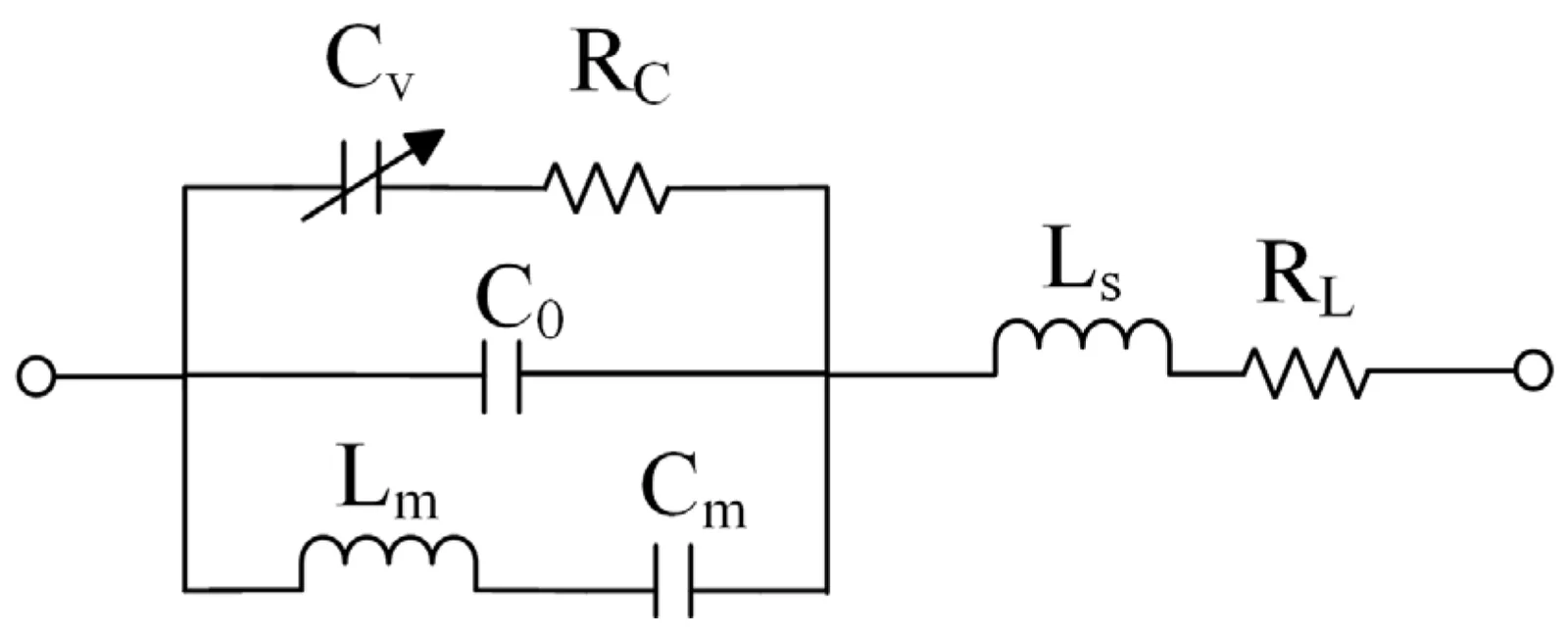
Reconfigurable AWLR module with lossy components.

In order to elaborate on the working principle of the tunable capacitor *C_v_*, a detailed theoretical analysis of the reconfigurable AWLR module is carried out. The impedance *Z*_1_ of the reconfigurable AWLR module can be mathematically expressed by (1). Consider that the series inductor *L_s_* and the shunt tunable capacitor *C_v_* are lossy components in practice. Figure 1a can be modified as shown in Figure 3. The parasitic resistances *R_c_* and *R_L_* can be calculated by (2) and (3), respectively. The impedance *Z*_1_ can be modified as *Z*_2_, expressed by (4).

(1) $$Z_{1}=\frac{1}{sC_{v}+sC_{0}+\left(\frac{1}{R_{m}+sL_{m}+\frac{1}{sC_{m}}}\right)}+sL_{s}$$

(2) $$R_{C}=1/\omega L_{C}Q_{C}$$

(3) $$R_{L}=\omega L/Q_{L}$$

(4) $$Z_{2}=\frac{1}{sC_{0}+\left(\frac{1}{\frac{1}{sC_{v}}+R_{C}}\right)+\left(\frac{1}{R_{m}+sL_{m}+\frac{1}{sC_{m}}}\right)}+sL_{s}+R_{L}$$

where *s* = *jω*, *Q_L_* and *Q_c_* are the quality factors of inductance and capacitance, respectively.

**Figure 4:**
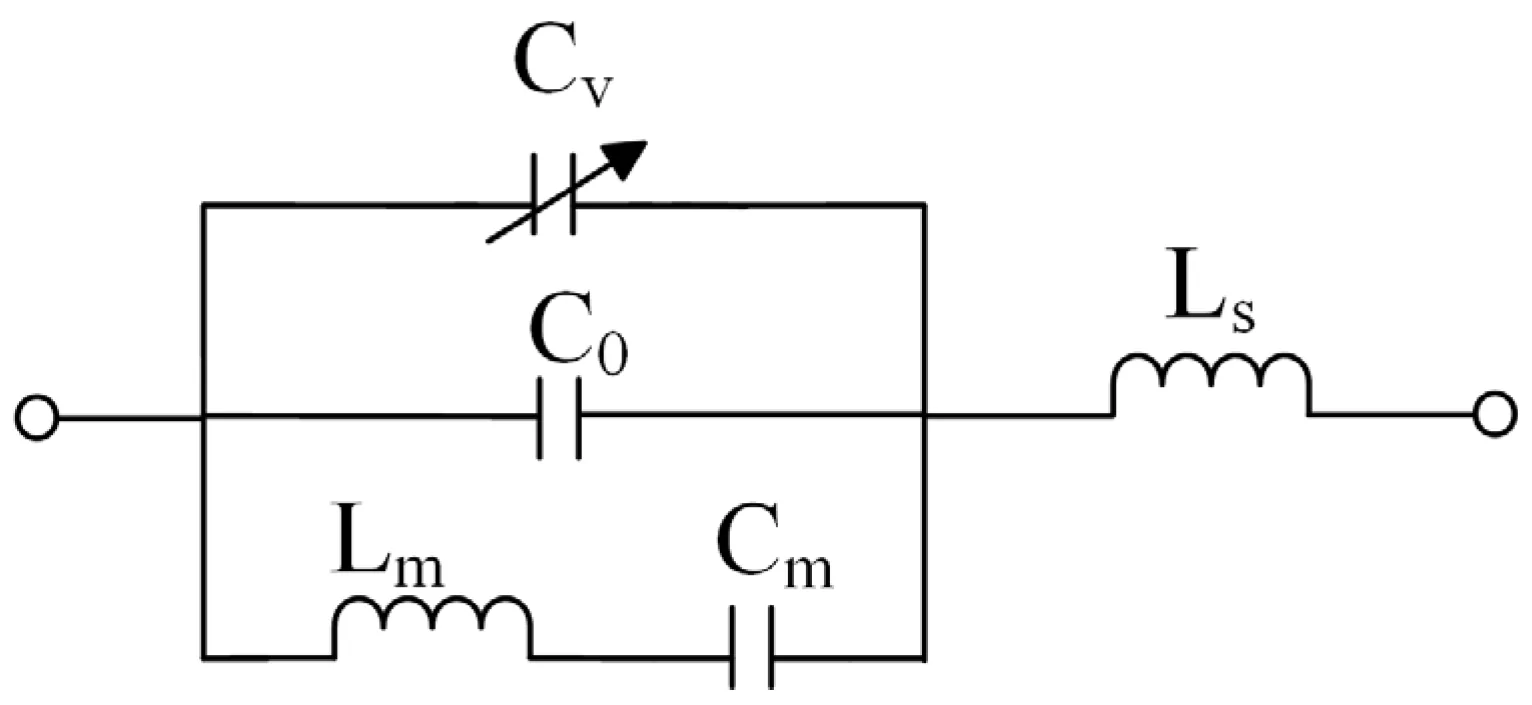
Ideal equivalent circuit of the reconfigurable AWLR module.

To simplify the analysis, an ideal AWLR model without the motional resistance *R_m_* is employed. The parasitic resistances *R_c_* and *R_L_* of the capacitor and inductor are removed, which can be regarded as a constant near the resonant frequency of the AWR. The structure shown in Figure 3 can be simplified as shown in Figure 4. The impedance *Z*_2_ can be modified as *Z*_3_, expressed by (5). When the denominator of the impedance *Z*_3_ is zero, the transmission zero *f_zero_* of the reconfigurable AWLR module can be calculated by (6). It is noted that the transmission zeros are determined by the tunable capacitor *C_v_* and are independent of the series inductor *L_s_*. Therefore, the location of the transmission zero can be controlled exclusively by adjusting the tunable capacitor. The analysis results are in good agreement with the simulated results of admittance in Figure 2. Figure 2 shows that the anti-resonant frequency is affected only by the tunable capacitance *C_v_*. When the numerator of the impedance *Z*_3_ is zero, the transmission pole of the reconfigurable AWLR module can be calculated by (7). The transmission pole *f_pole_* of the reconfigurable AWLR module is determined by both the tunable capacitance *C_v_* and the series inductor *L_s_*, which is also in agreement with the simulated results of admittance in Figure 2.

(5) $$Z_{3}=\frac{s^{4}L_{s}L_{m}C_{m}C_{v}+s^{4}L_{s}L_{m}C_{m}C_{0}+s^{2}L_{s}C_{v}+s^{2}L_{s}C_{0}+s^{2}L_{s}C_{m}+s^{2}L_{m}C_{m}+1}{s^{3}L_{m}C_{m}C_{v}+s^{3}L_{m}C_{m}C_{0}+sC_{v}+sC_{0}+sC_{m}}$$

(6) $$f_{zero}=\pm \frac{1}{2\pi }\sqrt{\frac{C_{v}+C_{0}+C_{m}}{L_{m}C_{m}\left(C_{v}+C_{0}\right)}}$$

(7) $$f_{pole}=\frac{1}{2\pi }\sqrt{\frac{L_{s}\left(C_{v}+C_{0}+C_{m}\right)+L_{m}C_{m}\pm \sqrt{{\left[L_{s}\left(C_{v}+C_{0}+C_{m}\right)+L_{m}C_{m}\right]}^{2}-4L_{s}L_{m}C_{m}\left(C_{v}+C_{0}\right)}}{2L_{s}L_{m}C_{m}\left(C_{v}+C_{0}\right)}}$$

**Figure 5:**
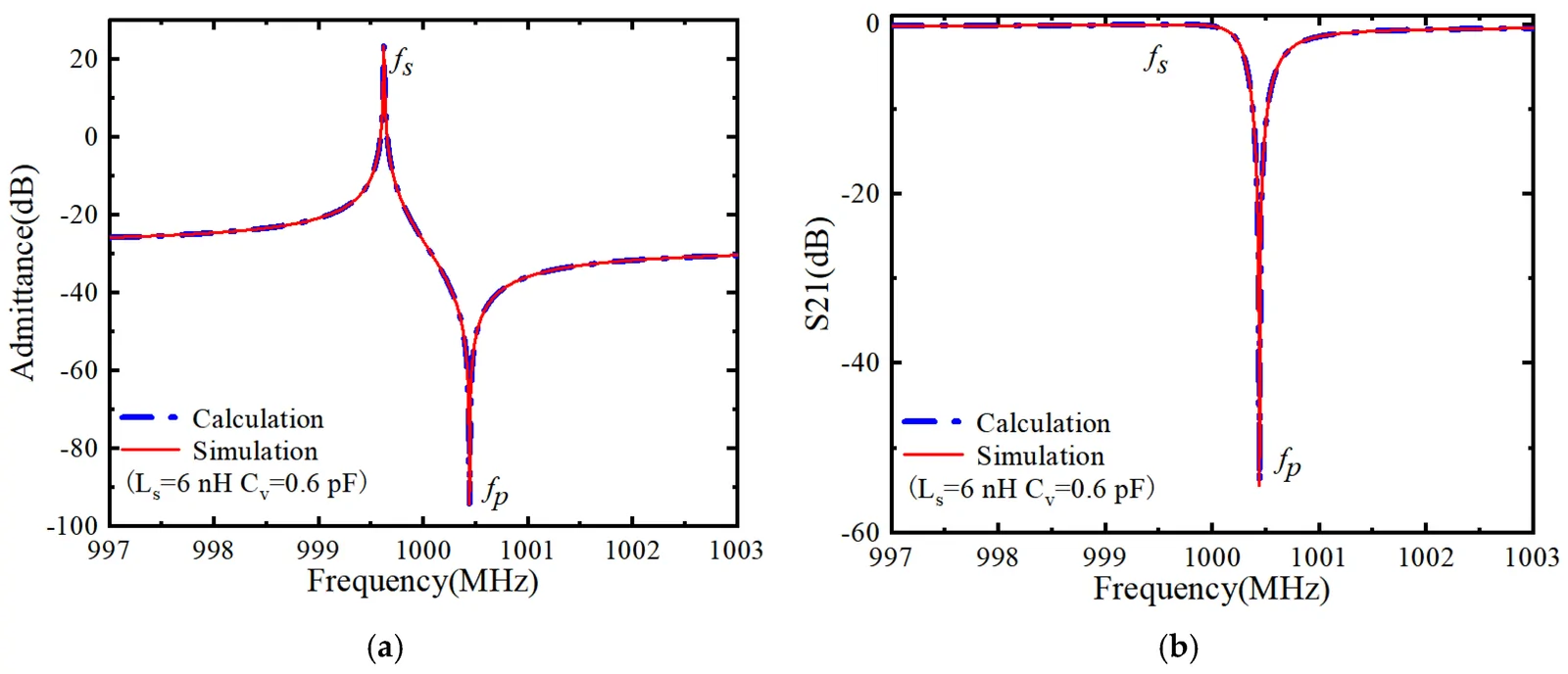
(**a**) Admittance response of the reconfigurable AWLR module; (**b**) frequency response of the reconfigurable AWLR module. (The values of *L_m_*, *C_m_*, *R_m_*, and *C*_0_ are 15.265 μH, 1.659 fF, 12.8 Ω, and 1.93 pF, respectively).

The S parameters of the reconfigurable AWLR module can be derived by employing the impedance *Z*_3_. It is expressed by (8). The value of *Z*_0_ is 50 Ω. The calculated results and simulated results of the admittance response (*Y* = 1/*Z_3_*) are shown in Figure 5a. The calculated and simulated results of the S parameters are shown in Figure 5b. The consistency results between the calculation and simulation confirm the correctness of the theoretical derivation.

(8) $$\left(\begin{array}{cc} S_{11} & S_{12}\\ S_{21} & S_{22} \end{array}\right)=\left(\begin{array}{cc} \frac{Z_{3}/Z_{0}}{2+Z_{3}/Z_{0}} & \frac{2}{2+Z_{3}/Z_{0}}\\ \frac{2}{2+Z_{3}/Z_{0}} & \frac{Z_{3}/Z_{0}}{2+Z_{3}/Z_{0}} \end{array}\right)$$

**Figure 6:**
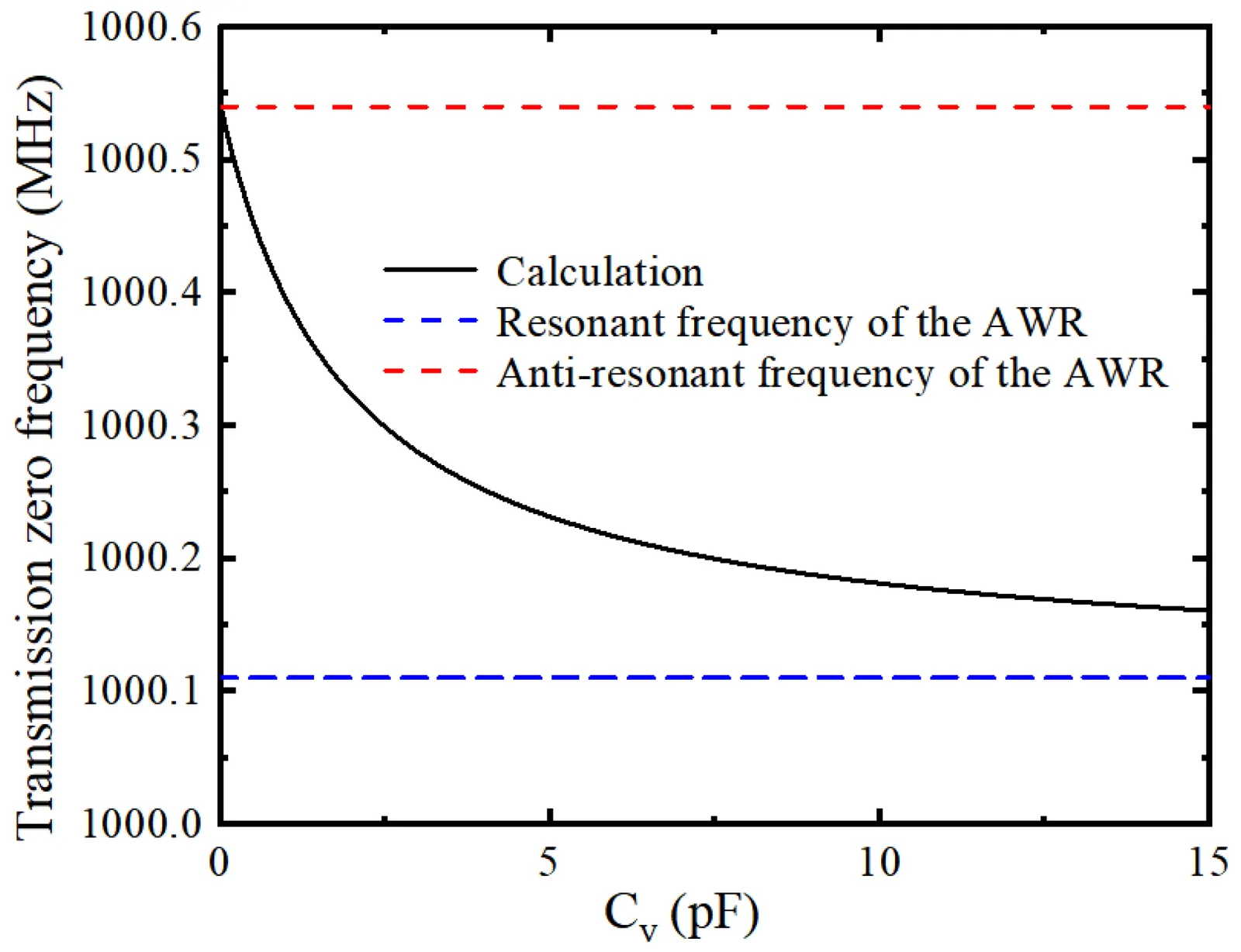
Relationship between the value of *C_v_* and the transmission zero frequency of the reconfigurable AWLR module.

The reconfigurable AWLR module is the core component for designing bandwidth-tunable AWLR-based filters. In the reconfigurable AWLR module, the value of the series inductance *L_s_* is fixed, and the tunable bandwidth is achieved by the tunable capacitance *C_v_*. Therefore, the capacitance range of the tunable capacitor is critical to the bandwidth tuning capability of the hybrid filter and directly determines the varactor diode selection. The relationship between the value of *C_v_* and the transmission zero *f_zero_* of the reconfigurable AWLR module can be calculated by (6). When the value of *C_v_* approaches infinity, the transmission zero frequency can be expressed by (9). It is exactly the resonant frequency of the AWR. Conversely, when the value of *C_v_* tends to zero, the transmission zero frequency can be expressed by (10). It corresponds to the anti-resonant frequency of the AWR. Therefore, the transmission zero frequency of the reconfigurable AWLR module can only be tuned between the resonant and anti-resonant frequencies of the AWR. The relationship between the value of *C_v_* and the transmission zero frequency of the reconfigurable AWLR module is plotted in Figure 6. The results is calculated by (6).

(9) $$\underset{C_{v}\to \infty }{\lim}f_{zero}=\frac{1}{2\pi \sqrt{L_{m}C_{m}}}$$

(10) $$\underset{C_{v}\to 0}{\lim}f_{zero}=\frac{1}{2\pi }\sqrt{\frac{C_{0}+C_{m}}{L_{m}C_{m}C_{0}}}$$

Figure 6 demonstrates that the transmission zero frequency of the reconfigurable AWLR module exhibits a strong dependence on the tunable capacitance range from 0 pF to 5 pF. The variation of the transmission zero frequency becomes marginal for capacitance values above 5 pF. In the subsequent design of the bandwidth-tunable hybrid filter, the reconfigurable AWLR module will be designed in the shunt branches of the ladder-type topology. The excessively large capacitance values are not desirable. As illustrated in Figure 2, both the series inductance and the tunable capacitance can affect the resonant frequency of the reconfigurable AWLR module. An overly large capacitance would render the resonant frequency ineffective in controlling the transmission zero of the hybrid filter. In addition, the capacitance variation that changes the transmission zero and pole may also affect the insertion loss of the reconfigurable hybrid filter.

**Figure 7:**
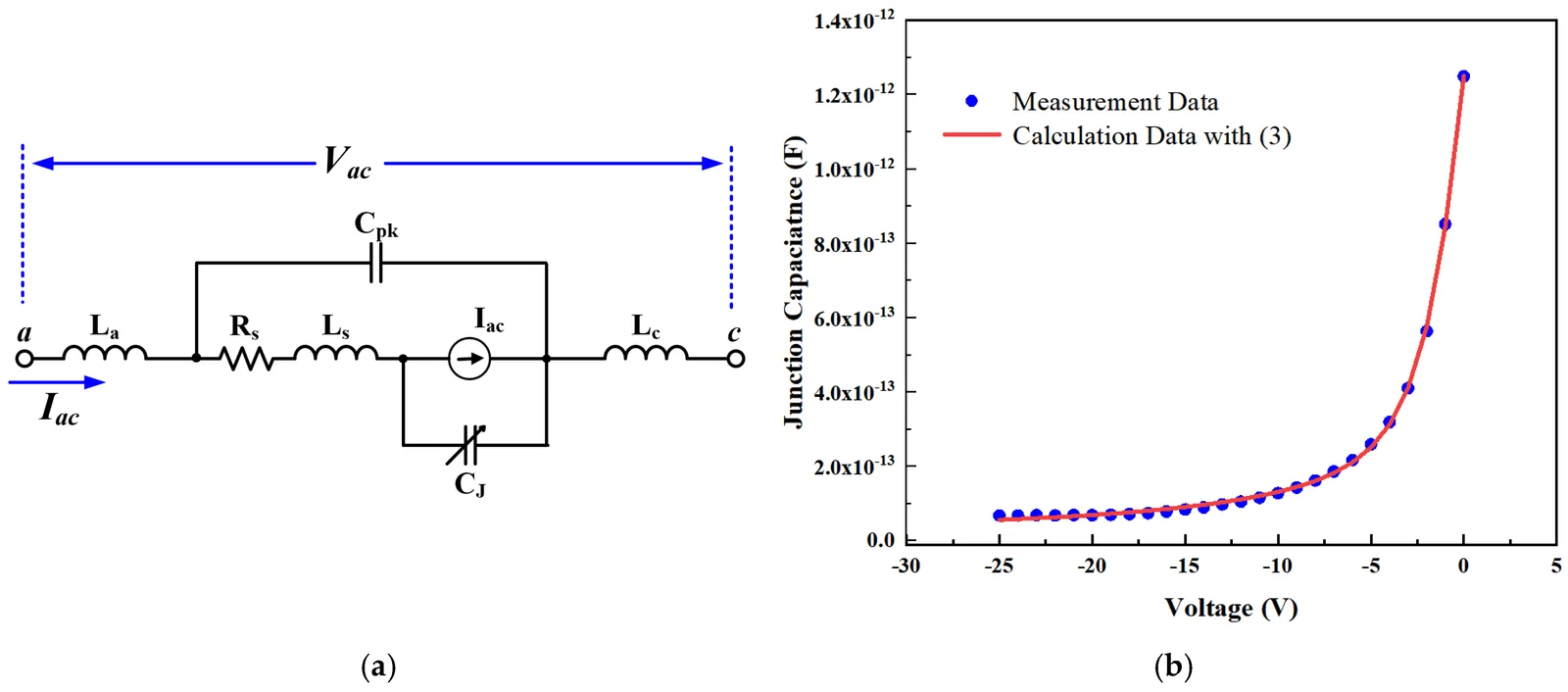
(**a**) Equivalent circuit model of the varactor diode; (**b**) verification of the junction capacitance of the varactor diode.

The tunable capacitance is finally selected to range from 0.1 pF to 1.2 pF. Correspondingly, the MA46H120 varactor (MACOM Technology Solutions Holdings, Inc., Lowell, MA, USA) diode is chosen to provide a capacitance tuning range from 0.1 pF to 1.2 pF. Its equivalent circuit and key parameters are reported in our previous work [32]. The equivalent circuit of the varactor diode is shown in Figure 7a. It is composed of five parasitic parameters *L_a_*, *L_c_*, *C_pk_*, *R_s_*, and *L_s_*, a current *I_ac_*, and a tunable capacitor *C_v_*. The tunable capacitor *C_v_* can be calculated by (11) [32]. The comparison between the measured and calculated data of the varactor diode’s capacitance is shown in Figure 7b. The consistency results validate the correctness of the analytical derivation.

(11) $$C_{v}=\frac{C_{J0}}{{\left(1+\frac{V_{ac}}{V_{bi}}\right)}^{\left(p_{1}+p_{2}\left(1+\tanh\left(p_{3}-p_{4}V_{ac}\right)\right)\right)}}$$

where *C_J_*_0_ is the reference capacitance, *V_bi_* is the built-in voltage, and *p*_1_, *p*_2_, and *p*_3_ are the fitting parameters.

### 2.2. Design and Analysis of Reconfigurable AWLR-Based Filters

**Figure 8:**
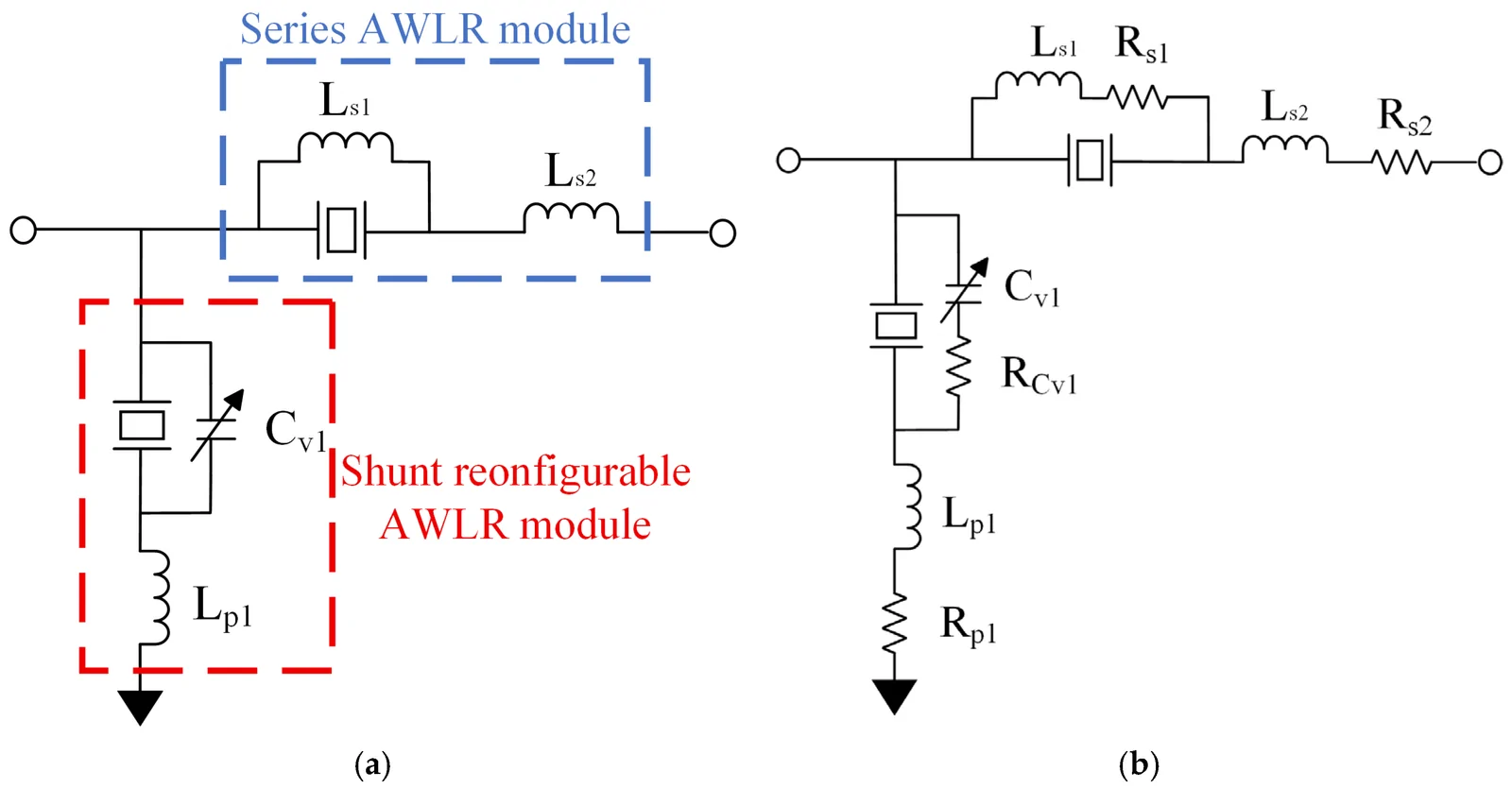
(**a**) Circuit configuration of the reconfigurable AWLR-based hybrid ladder filter; (**b**) circuit configuration of the reconfigurable AWLR-based hybrid ladder filter including lumped-element losses.

The proposed reconfigurable AWLR-based filter is presented in Figure 8a. It is a ladder-type structure. The series AWLR module is made up of an AWR and two inductors *L_s_*_1_ and *L_s_*_2_. The shunt reconfigurable AWLR module consists of an AWR, an inductor *L_p_*_1_, and a tunable capacitor *C_v_*_1_. In the reconfigurable AWLR-based filter, the employed AWRs have the same resonant frequency and impedance. The series AWLR module and the shunt reconfigurable AWLR module employed inductors and capacitors to flexibly adjust the resonant and anti-resonant frequencies of the AWLR module, thereby controlling the locations of the transmission zeros and poles. They jointly constitute the reconfigurable hybrid bandpass filter. To more accurately evaluate the insertion loss and bandwidth tuning range of the reconfigurable hybrid filter using analytical expressions, the losses of the capacitors and inductors are taken into account. The circuit structure in Figure 8a can be modified to that shown in Figure 8b.

The impedance of the series AWLR module can be calculated by (12). According to expression (4), the impedance of the shunt-reconfigurable AWLR module can be calculated by (13). The overall ABCD matrix of the reconfigurable hybrid filter is obtained by cascading the ABCD matrices of the individual series and shunt branches, as expressed in (14)–(16). The transfer function of the reconfigurable hybrid filter is derived from the ABCD matrix and is expressed as *S*_21_ in (17).

(12) $$Z_{s1}=\frac{1}{sC_{0}+\left(\frac{1}{sL_{s1}+R_{s1}}\right)+\left(\frac{1}{R_{m}+sL_{m}+\frac{1}{sC_{m}}}\right)}+sL_{s2}+R_{s2}$$

(13) $$Z_{p1}=\frac{1}{sC_{0}+\left(\frac{1}{\frac{1}{sC_{v1}}+R_{C_{v1}}}\right)+\left(\frac{1}{R_{m}+sL_{m}+\frac{1}{sC_{m}}}\right)}+sL_{p1}+R_{p1}$$

(14) $${\left(\begin{array}{cc} A & B\\ C & D \end{array}\right)}_{series}=\left(\begin{array}{cc} 1 & Z_{s1}\\ 0 & 1 \end{array}\right)$$

(15) $${\left(\begin{array}{cc} A & B\\ C & D \end{array}\right)}_{shunt}=\left(\begin{array}{cc} 1 & 0\\ 1/Z_{p1} & 1 \end{array}\right)$$

(16) $${\left(\begin{array}{cc} A & B\\ C & D \end{array}\right)}_{total}={\left(\begin{array}{cc} A & B\\ C & D \end{array}\right)}_{series}\times {\left(\begin{array}{cc} A & B\\ C & D \end{array}\right)}_{shunt}=\left(\begin{array}{cc} 1+Z_{s1}/Z_{p1} & Z_{s1}\\ 1/Z_{p1} & 1 \end{array}\right)$$

(17) $$S_{21}=\frac{2}{2+\frac{Z_{s1}}{Z_{p1}}+\frac{Z_{s1}}{Z_{0}}+\frac{Z_{0}}{Z_{p1}}}$$

**Figure 9:**
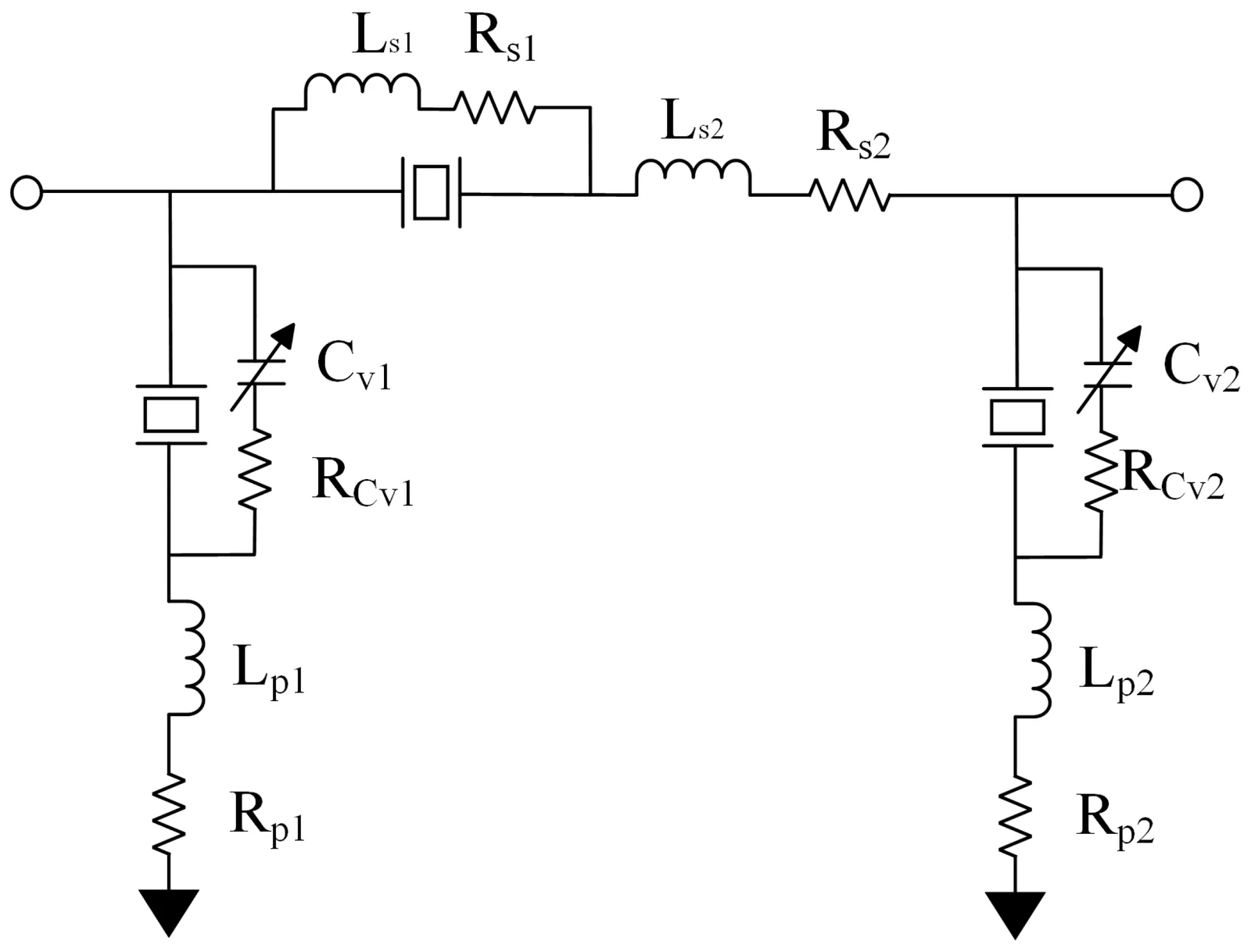
Circuit configuration of the reconfigurable AWLR-based hybrid with higher-order topology.

The above analysis has focused on the single-series and single-shunt configuration in Figure 8. The proposed reconfigurable hybrid filter can be extended to higher-order stages by increasing the number of shunt or series branches. A single series and two-shunt configuration of the reconfigurable hybrid filter is presented in Figure 9. To distinguish between the two reconfigurable hybrid filters shown in Figure 8 and Figure 9, they are named Type I and Type II, respectively. In the reconfigurable hybrid filter of Type II, the impedance *Z_p_*_2_ of the additional shunt branch can be calculated by (18). The overall ABCD matrix and the corresponding transfer function *S*_21_ of the reconfigurable hybrid filter can be derived from expressions (19)–(21).

(18) $$Z_{p2}=\frac{1}{sC_{0}+\left(\frac{1}{\frac{1}{sC_{v2}}+R_{C_{v2}}}\right)+\left(\frac{1}{R_{m}+sL_{m}+\frac{1}{sC_{m}}}\right)}+sL_{p2}+R_{p2}$$

(19) $${\left(\begin{array}{cc} A & B\\ C & D \end{array}\right)}_{total}={\left(\begin{array}{cc} A & B\\ C & D \end{array}\right)}_{shunt1}{\left(\begin{array}{cc} A & B\\ C & D \end{array}\right)}_{series}\times {\left(\begin{array}{cc} A & B\\ C & D \end{array}\right)}_{shunt2}$$

(20) $${\left(\begin{array}{cc} A & B\\ C & D \end{array}\right)}_{total}=\left(\begin{array}{cc} 1+\frac{Z_{s1}}{Z_{p2}} & Z_{s1}\\ \frac{1}{Z_{p1}}+\frac{1}{Z_{p2}}+\frac{Z_{s1}}{Z_{p1}Z_{p2}} & 1+\frac{Z_{s1}}{Z_{p1}} \end{array}\right)$$

(21) $$S_{21}=\frac{2}{2+Z_{s1}\left(\frac{1}{Z_{p1}}+\frac{1}{Z_{p2}}\right)+\frac{Z_{s1}}{Z_{0}}+Z_{0}\left(\frac{1}{Z_{p1}}+\frac{1}{Z_{p2}}+\frac{Z_{s1}}{Z_{p1}Z_{p2}}\right)}$$

Using expressions (17) and (21), the insertion loss and bandwidth of the two reconfigurable hybrid filters shown in Figure 8 and Figure 9 can be directly calculated. This approach enables theoretical prediction of the electrical performance and facilitates the trade-off between insertion loss and bandwidth tuning range.

### 2.3. Calculation and Analysis of Insertion Loss and Bandwidth Tuning

Based on the analytical expression derived in Section 2.2, the insertion loss and 3 dB fractional bandwidth of the Type I reconfigurable hybrid filter in Figure 8 are calculated. They can be directly calculated by using expression (17) with the MATLAB program (2025). The BVD parameters of the commercial TC0528A SAW resonator are used for the calculation, as summarized in Table 1. The value of the inductors employed in the reconfigurable hybrid filter is empirically selected according to the fixed-bandwidth filters reported in our previous works [23,24,25]. They are included in Table 2. The parasitic resistances of those inductors are calculated based on a quality factor of 30 by (3). The tunable capacitor, ranging from 0.1 pF to 1.2 pF, is realized by the MA46H120 varactor diode described in Section 2.1. Its parasitic resistance is 1.15 Ω.

**Figure 10:**
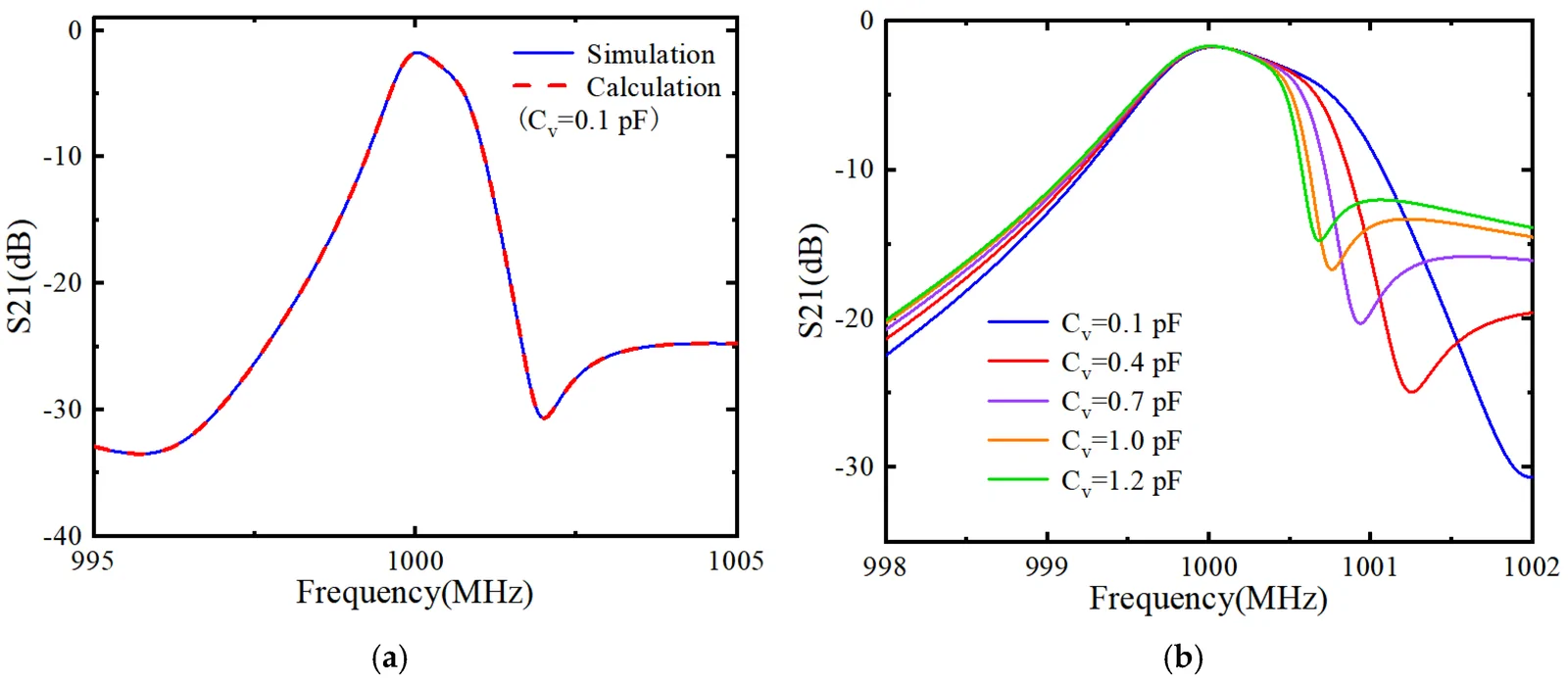
(**a**) Comparison between calculated and simulated *S*_21_ responses (Type I); (**b**) calculated frequency responses of the hybrid filter for various tunable capacitance values (Type I).

The frequency response of the Type I reconfigurable AWLR-based filter obtained from analytical calculation and circuit simulation is presented in Figure 10a. The good agreement between the two results validates the correctness of the theoretical derivation. The value of the tunable capacitor is 0.1 pF in Figure 10a. The calculated frequency responses under different capacitance values are shown in Figure 10b. It can be observed that the bandwidth narrows as the value of the tunable capacitor increases. This verifies the continuous tunability of bandwidth for the reconfigurable hybrid filter.

**Figure 11:**
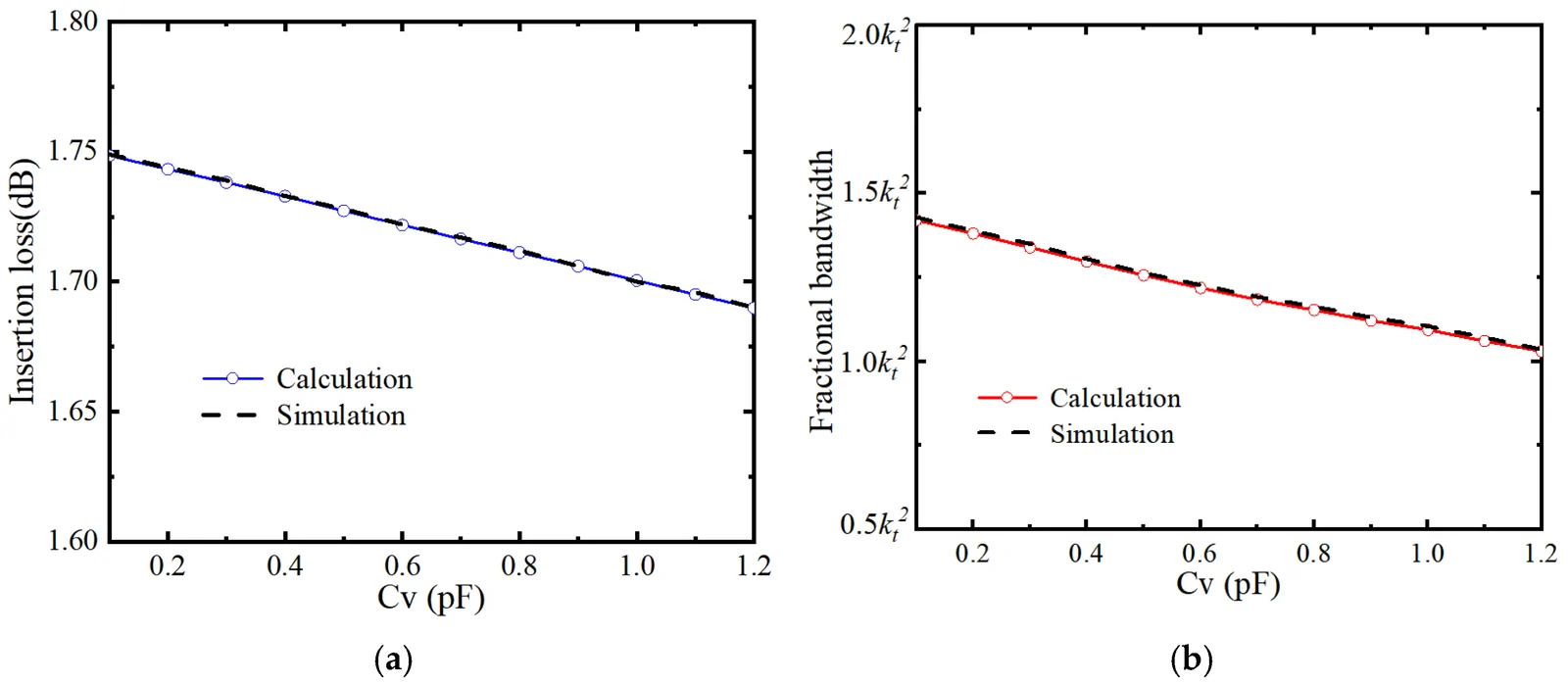
(**a**) Calculated insertion loss of the reconfigurable hybrid filter (Type I); (**b**) calculated fractional bandwidth of the reconfigurable hybrid filter (Type I).

The calculated and simulated insertion loss and 3 dB fractional bandwidth of the reconfigurable hybrid filters for different tunable capacitance values are presented in Figure 11a and Figure 11b, respectively. Both of them show good agreement between the calculated and simulated results. As shown in Figure 11a, the insertion loss remains below 1.75 dB over the capacitance range from 0.1 pF to 1.2 pF. Figure 11b indicates that the 3 dB fractional bandwidth can be tuned from 1.01*k_t_*^2^ to 1.46*k_t_*^2^ as *C_v_* varies from 0.1 pF to 1.2 pF. The calculated electromechanical coupling coefficient *k_t_*^2^ (*k_t_*^2^ = 1 − *ω_s_*^2^/*ω_p_*^2^) of the SAW BVD model is 0.085%. The results demonstrate that the fractional bandwidth achieves a 45% improvement over the minimum fractional bandwidth, while the insertion loss is kept below 1.75 dB.

**Figure 12:**
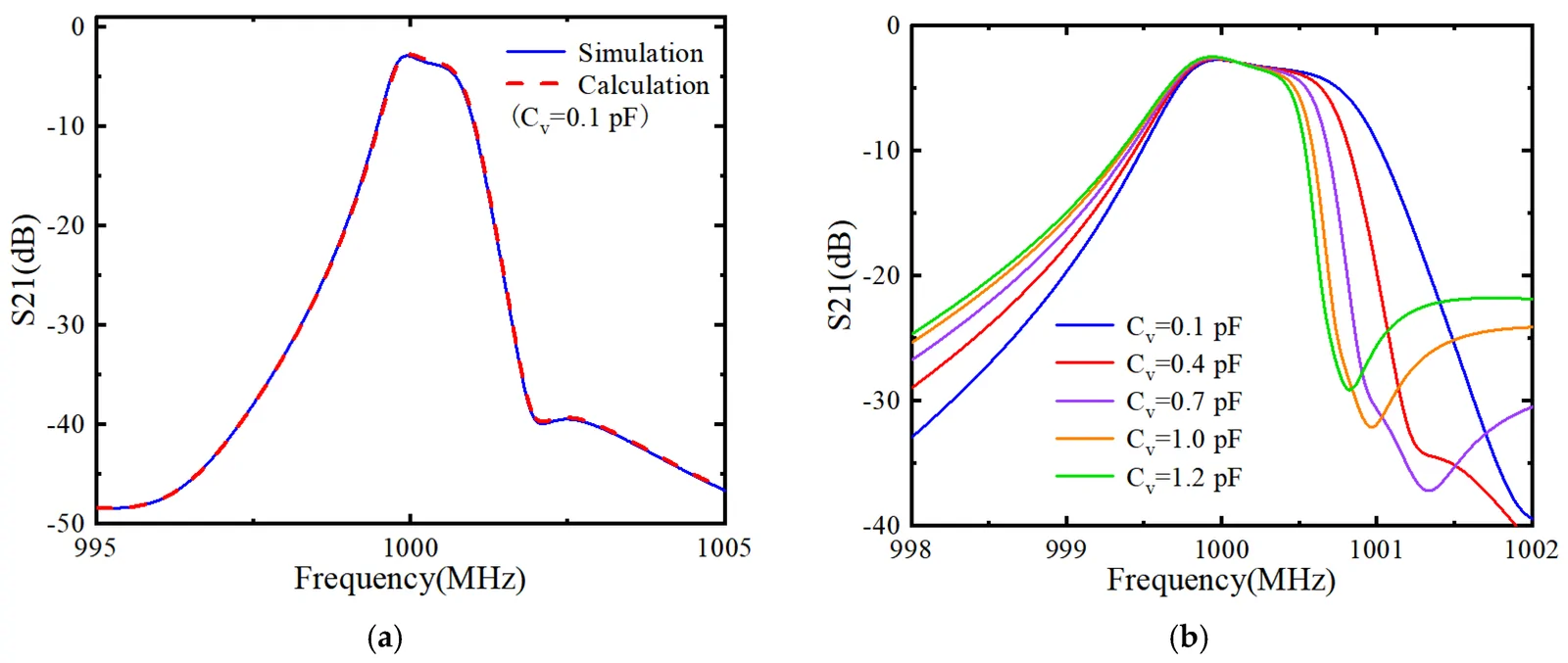
(**a**) Comparison between calculated and simulated *S*_21_ responses (Type II); (**b**) calculated frequency responses of the hybrid filter for various tunable capacitance values (Type II).

Based on the Type II reconfigurable hybrid filter in Figure 9, its frequency response can be calculated by expression (21). The inductor *L_p_*_2_ shown in Figure 9 is set to 13 nH. The remaining circuit parameters of the reconfigurable hybrid filter in Figure 9 are presented in Table 1 and Table 2. The parasitic resistance of inductors is calculated under a quality factor of 30 by (3). Figure 12a compares the calculated and simulated frequency responses when the value of the tunable capacitor is 0.1 pF. Compared with the results of the Type I reconfigurable hybrid filter, it exhibits improved out-of-band rejection at the cost of a slight increase in insertion loss. Figure 12b presents the calculated frequency responses for different *C_v_* values. It is noted that the Type II reconfigurable hybrid filter also achieves effective bandwidth tunability.

**Figure 13:**
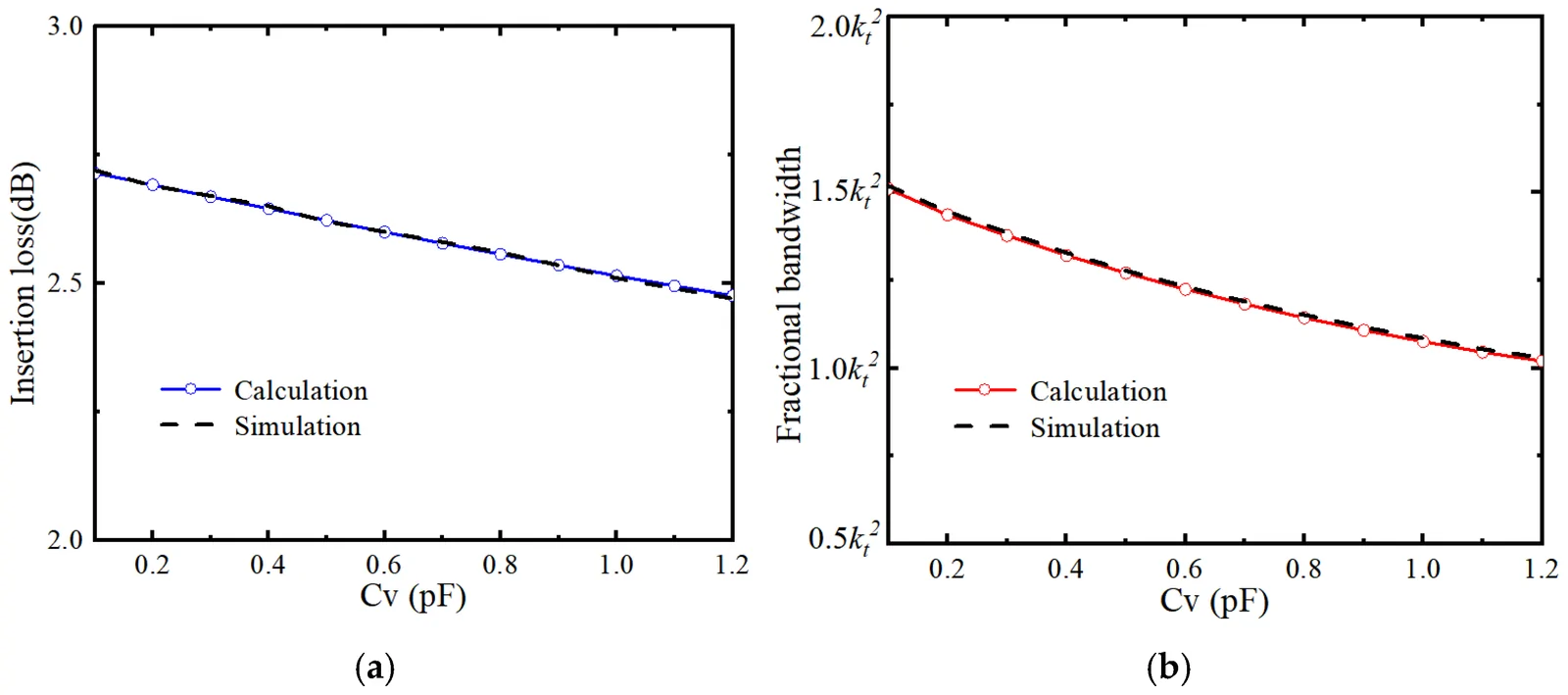
(**a**) Calculated insertion loss (Type II); (**b**) calculated fractional bandwidth of the reconfigurable hybrid filter (Type II).

Figure 13a,b present the calculated and simulated insertion loss and 3 dB fractional bandwidth of the Type II hybrid filter under different *C_v_* values, respectively. Good agreement is observed between the calculated and simulated results in Figure 13a,b. The insertion loss remains below 2.75 dB over the entire tuning range, while the fractional bandwidth is continuously tunable from 1.01*k_t_*^2^ to 1.56*k_t_*^2^ as *C_v_* increases from 0.1 pF to 1.2 pF.

Based on the theoretical analysis of the two-type hybrid filter configurations, the proposed reconfigurable hybrid filters can achieve bandwidth tunability while effectively controlling insertion loss. The analytical expressions in (17) and (21) allow direct prediction of the filter’s electrical characteristics, thus enabling the achievement of a preferable filter configuration. Their experimental verification will be carried out in Section 3.

## 3. Experimental Validation

**Figure 14:**
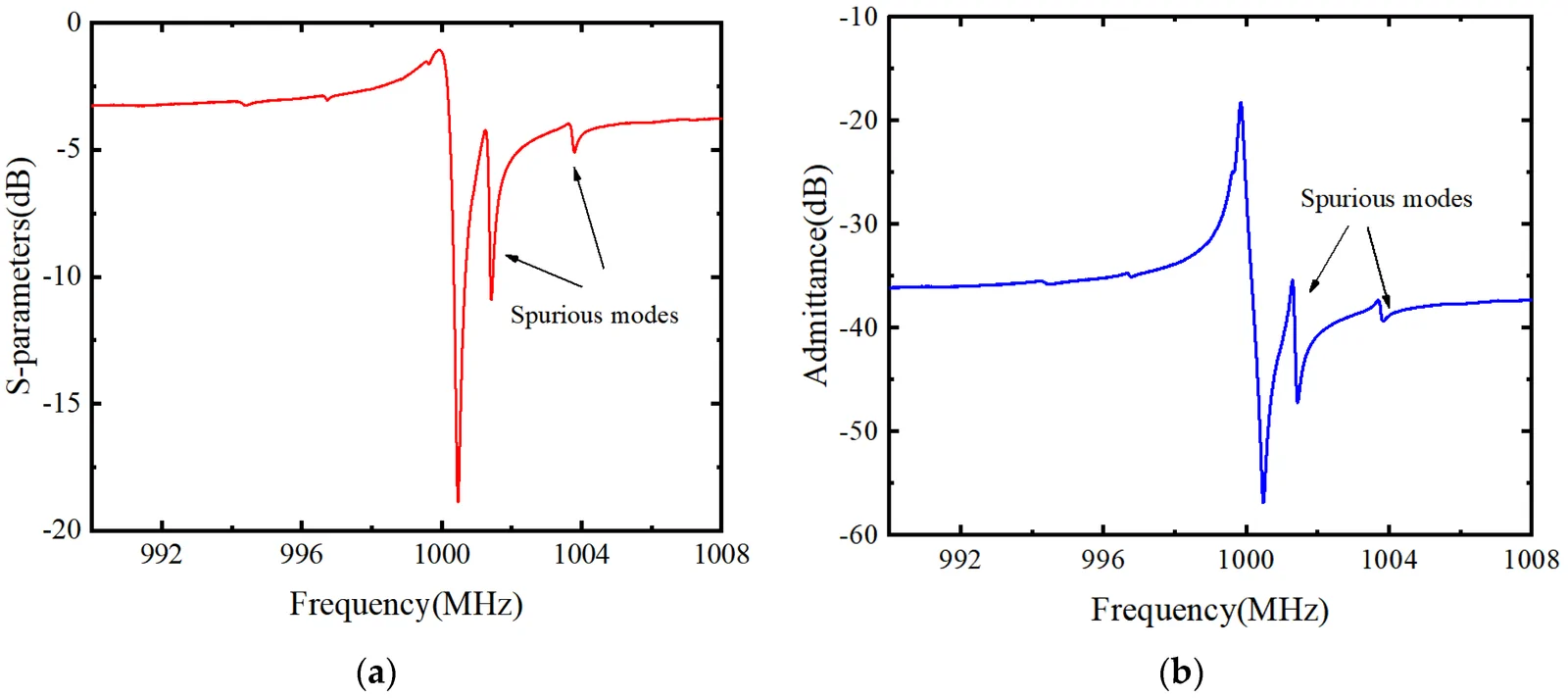
(**a**) Measured S-parameters of the SAW resonator (TC0528A); (**b**) measured admittance response of the SAW resonator (TC0528A).

To validate the theoretical analysis results presented in Section 2, the proposed two prototypes of Type I and Type II filters are fabricated and measured. Their circuit configurations are presented in Figure 8 and Figure 9, respectively. Both prototypes are based on the commercially available TC0528A SAW resonator (TAI-SAW, Taoyuan 324, Taiwan) and the MA46H120 varactor diode for tunable capacitance. The lumped inductors are implemented using surface-mount devices (SMDs) from Coilcraft (Cary, NC, USA). The two reconfigurable AWLR-based filters are fabricated on a Rogers RO4350B (Toronto, ON, Canada) substrate with the following parameters: relative dielectric permittivity *ε_r_* = 3.48, substrate thickness *H* = 0.508 mm, and dielectric loss tangent tan(*δ*) = 0.004. The simulation is performed in the Advanced Design System (ADS) software (2025). The measured S-parameters and admittance response of the commercial TC0528A SAW resonator are shown in Figure 14a and Figure 14b, respectively. The electromechanical coupling coefficient *k_t_*^2^ of the SAW resonator is 0.08%. It is noted that the measured results of the SAW resonator include the main resonance and the spurious modes. To ensure the accuracy of the filter simulations, the measured SNP data of the SAW resonator are directly used in the ADS circuit simulations to replace the BVD model. The equivalent circuit model of the varactor diode shown in Section 2.1 is used to replace the tunable capacitor. The measured RF characteristics of the SAW resonator and two filters are obtained using the Vector Network Analyzer (Keysight E5071C, Keysight, Santa Rosa, CA, USA) with the TRL and SOLT calibrations, respectively [33].

### 3.1. Reconfigurable AWLR-Based Filter of Type I

**Figure 15:**
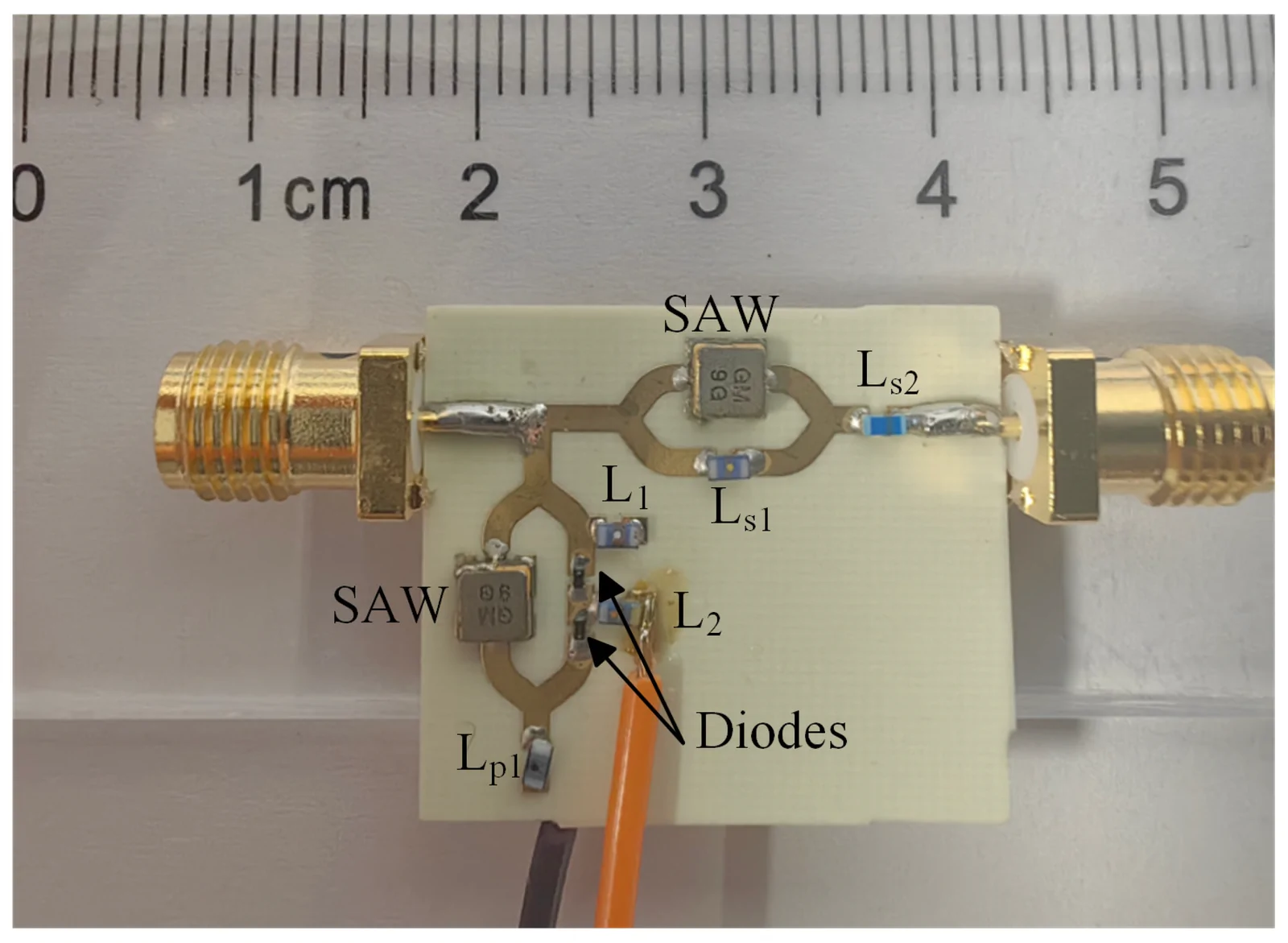
Manufactured prototype of Type I filter. (The values of *L_s_*_1_, *L_s_*_2_, *L_p_*_1_, *L*_1_, and *L*_2_ are 8.2 nH, 1.6 nH, 6.8 nH, 33 nH, and 8.2 nH, respectively).

The reconfigurable AWLR-based filter of Type I has been manufactured. Its manufactured prototype is shown in Figure 15. The inductance values are also presented in Figure 15. The diodes correspond to the MA46H120 varactor diode. To enhance the capacitance tuning range, two varactor diodes are connected in a back-to-back configuration. It can effectively increase the tuning ratio between the maximum and minimum capacitance. The inductor *L*_1_ in Figure 15 is the choke inductor, providing a low-impedance DC path and a high-impedance RF path. The inductor *L_p_*_1_ is employed to provide a DC path to ground. Two DC blocks are employed, placed in the cables between the device and the vector network analyzer to prevent DC signals from reaching the instrument.

**Figure 16:**
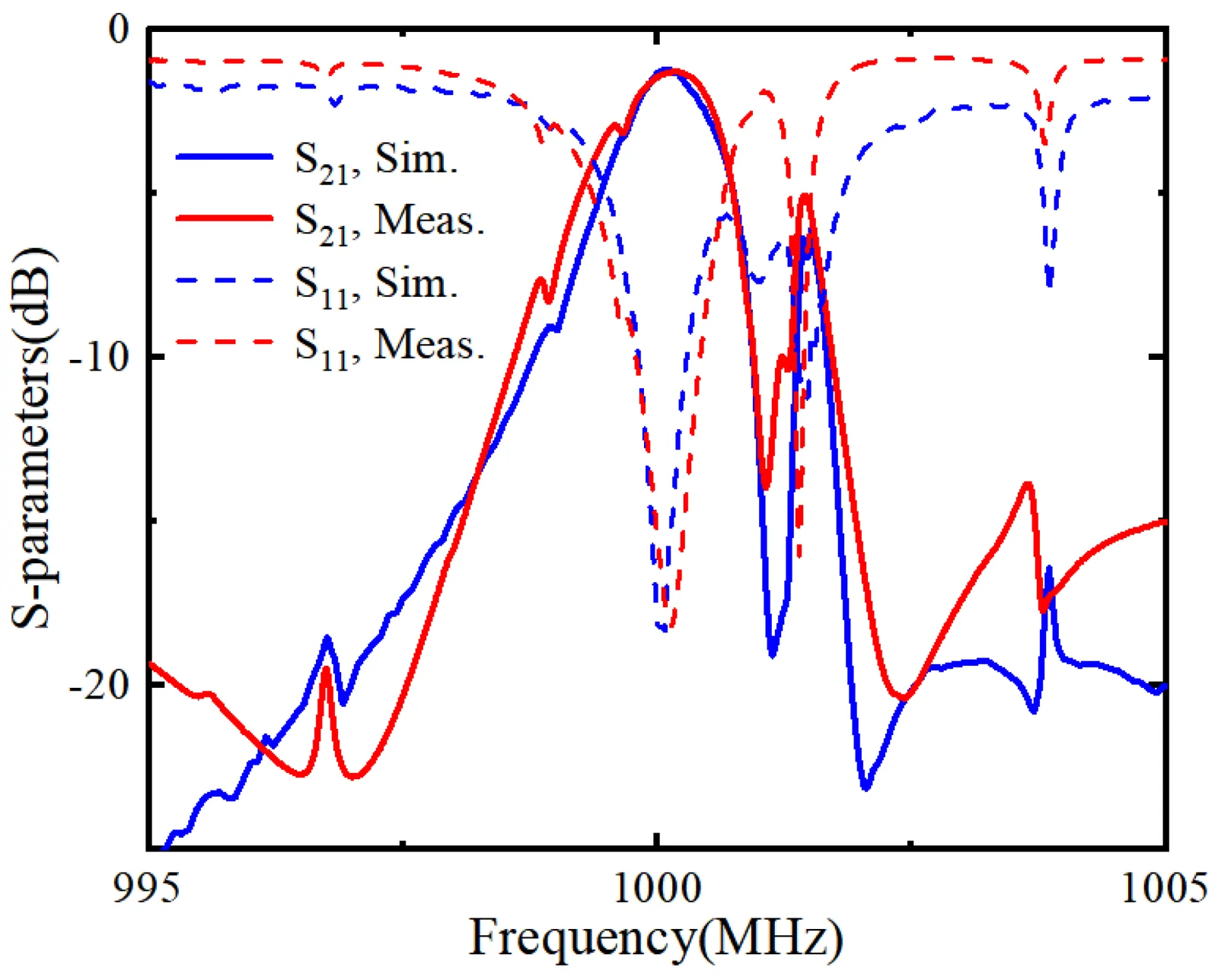
Simulated and measured S-parameters of the Type I filter.

The measured and simulated results of the Type I filter are plotted in Figure 16. The results are obtained when the varactor diode works at −8 V. A fair agreement is achieved between the RF-simulated and RF-measured S-parameters. The ripples in the transition band of the Type I filter are caused by the spurious modes of the SAW resonator, as illustrated in Figure 14. It is also reported in [6,21].

**Figure 17:**
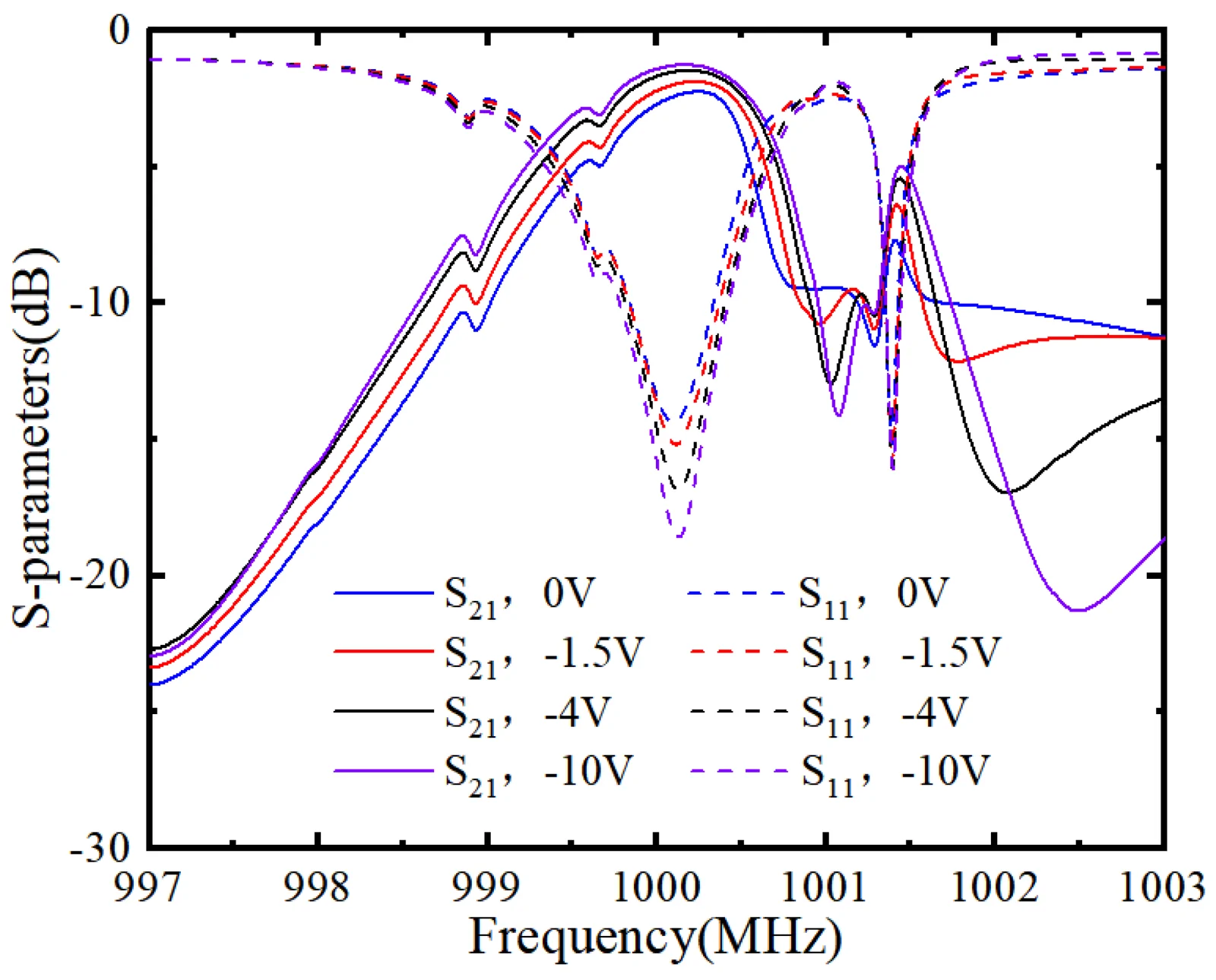
Measured S-parameters of the Type I filter under different varactor bias voltages.

**Figure 18:**
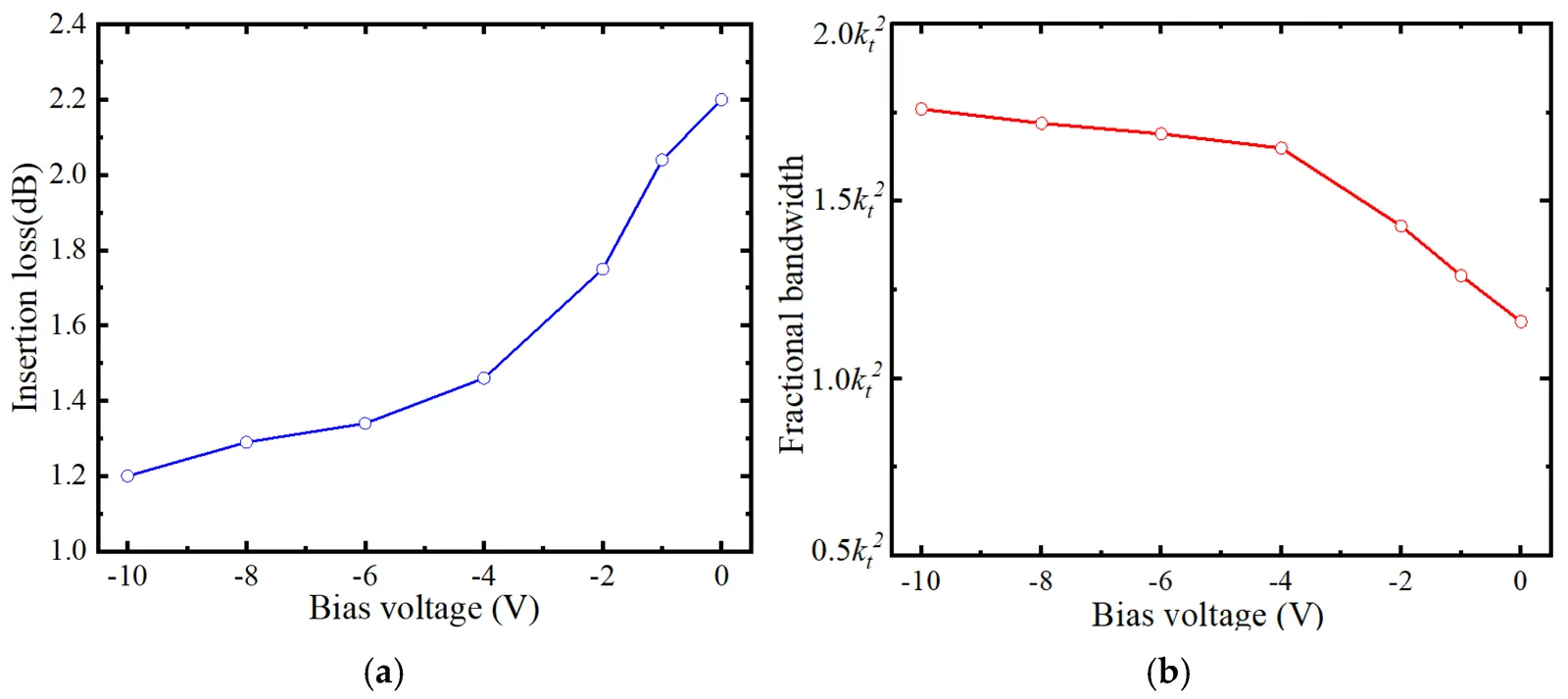
(**a**) Measured insertion loss (Type I); (**b**) measured fractional bandwidth of the reconfigurable hybrid filter (Type I).

The measured frequency responses of the Type I reconfigurable filter under different varactor bias voltages are shown in Figure 17. Figure 18a,b present the measured insertion loss and 3 dB fractional bandwidth under different varactor bias voltages, respectively. As the bias voltage increases from 0 V to −10 V, the junction capacitance is reduced, and the filter response consequently shifts toward a wider passband. The insertion loss increases from 1.2 dB to 2.2 dB, while the 3 dB fractional bandwidth expands from 1.16*k_t_*^2^ to 1.76*k_t_*^2^, corresponding to an absolute bandwidth increase from 0.93 MHz to 1.41 MHz. The 3 dB fractional bandwidth presents an enhancement of 51.7%. The results indicate that the Type I AWLR-based filter achieves continuous bandwidth tunability with low insertion loss. The observed trade-off between bandwidth and insertion loss is consistent with the theoretical predictions in Section 2. It validates the success of the proposed design methodology.

### 3.2. Reconfigurable AWLR-Based Filter of Type II

**Figure 19:**
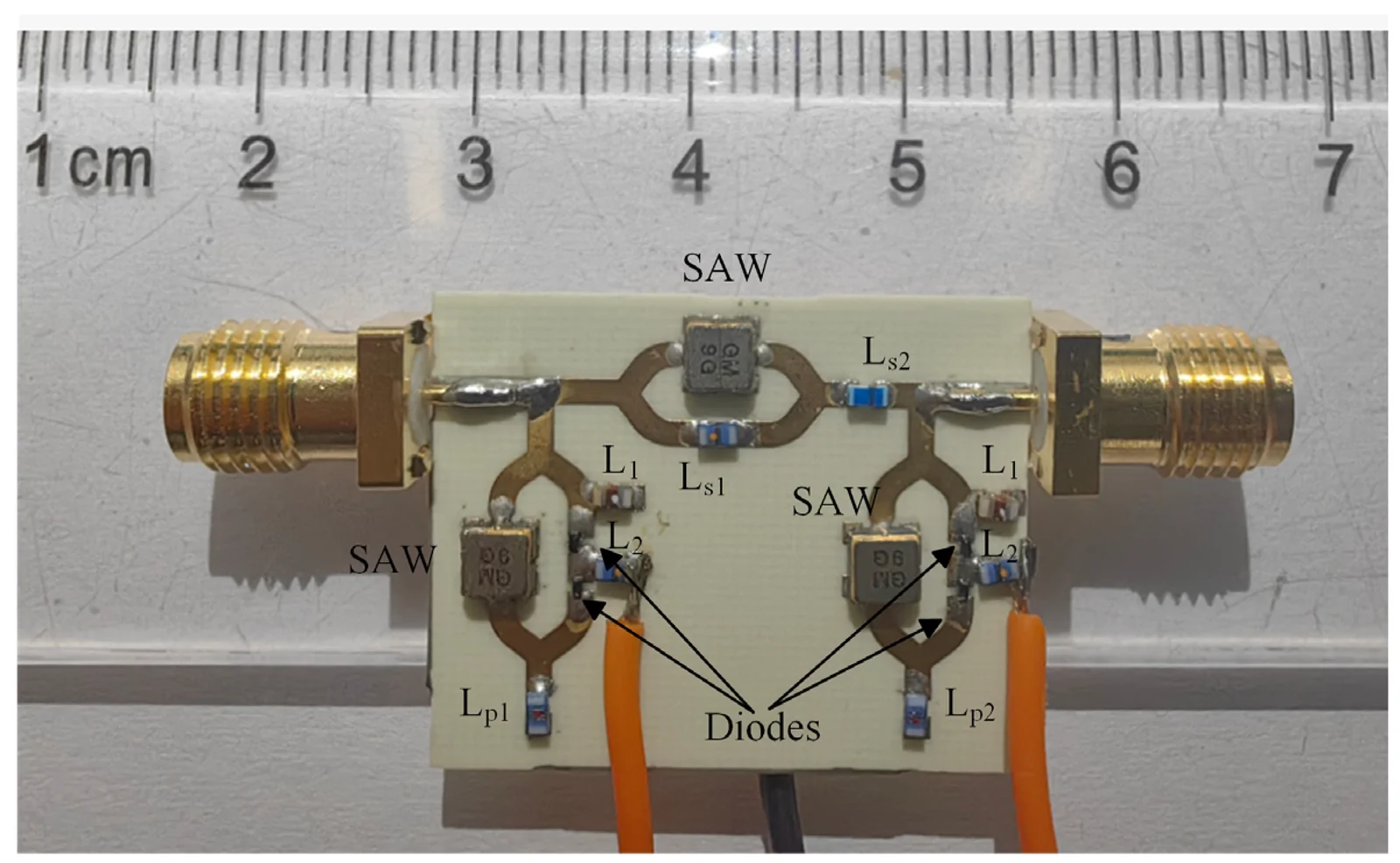
Manufactured prototype of Type II filter. (The values of *L_s_*_1_, *L_s_*_2_, *L_p_*_1_, *L_p_*_2_, *L*_1_, and *L*_2_ are 8.7 nH, 1.6 nH, 5.6 nH, 6.8 nH, 30 nH, and 9.5 nH, respectively).

**Figure 20:**
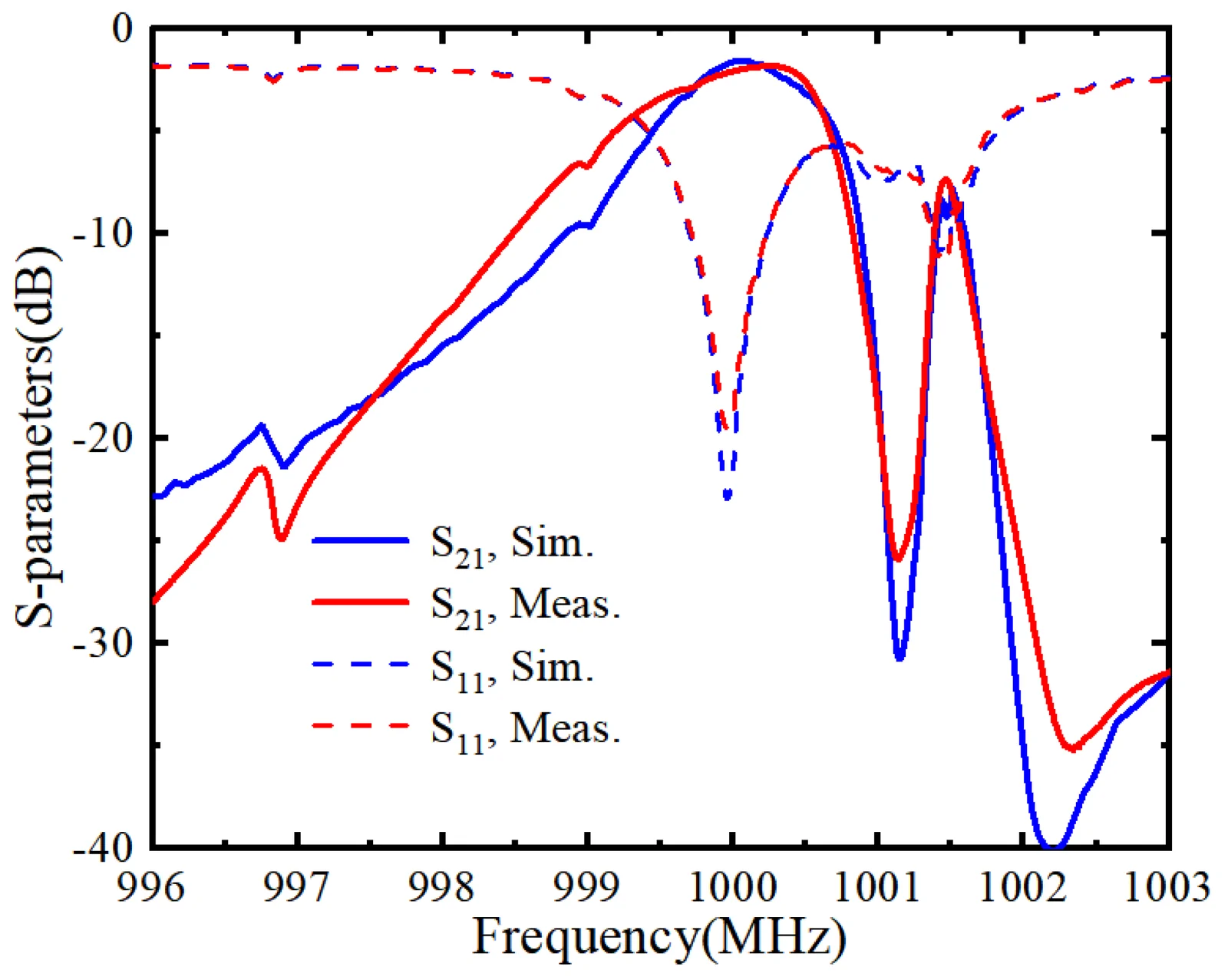
Simulated and measured S-parameters of the Type II filter.

The Type II reconfigurable AWLR-based filter has also been fabricated. Its prototype is shown in Figure 19. The corresponding inductance values are also presented in Figure 18. Compared with the Type I filter, it has an additional shunt AWLR reconfigurable module to realize the double-shunt configuration. Figure 20 presents the simulated and measured S-parameters of the Type II filters at the bias voltage of −8 V. Good agreement is observed between the two results. This confirms the accuracy of the simulation results.

**Figure 21:**
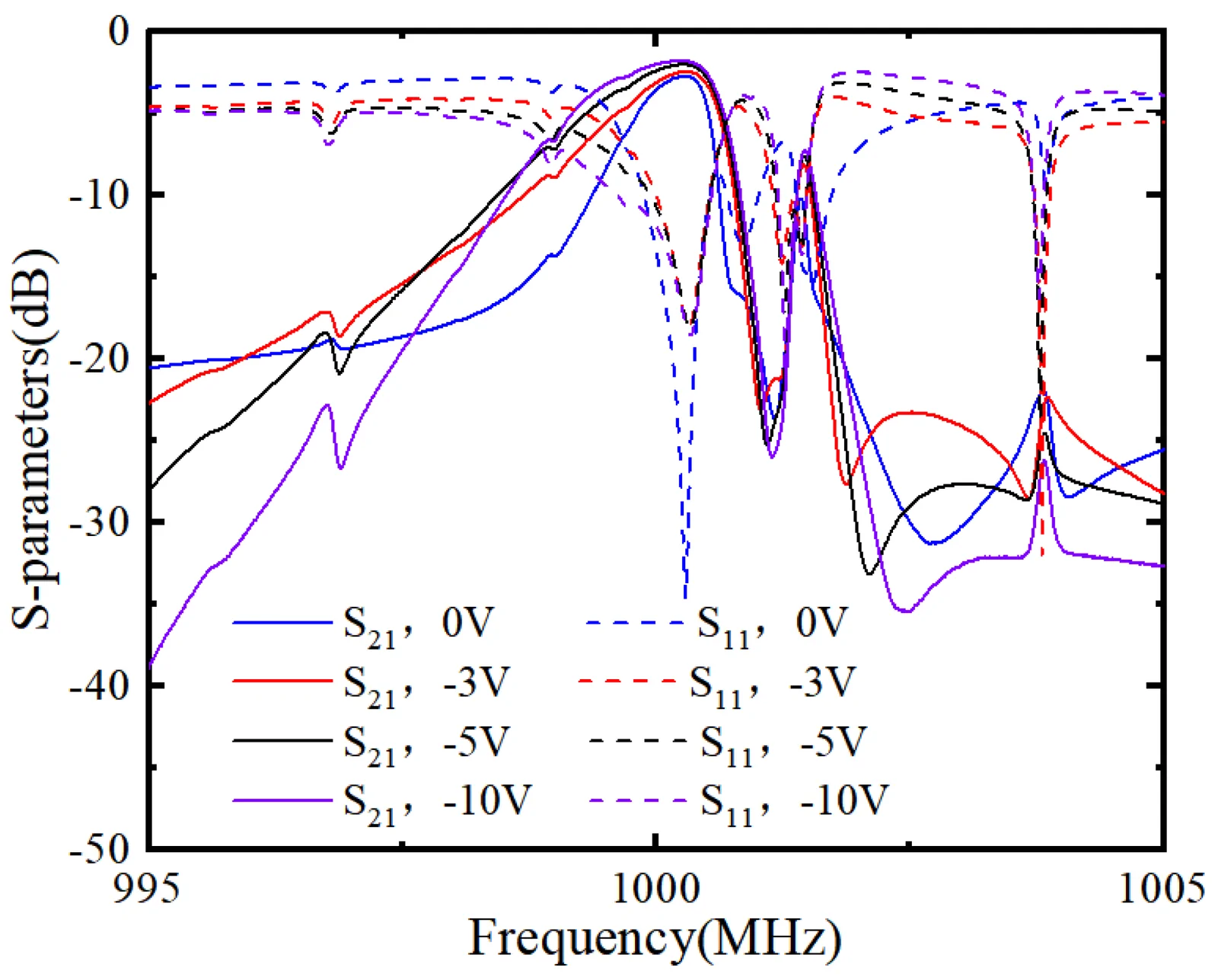
Measured S-parameters of the Type II filter under different varactor bias voltages.

**Figure 22:**
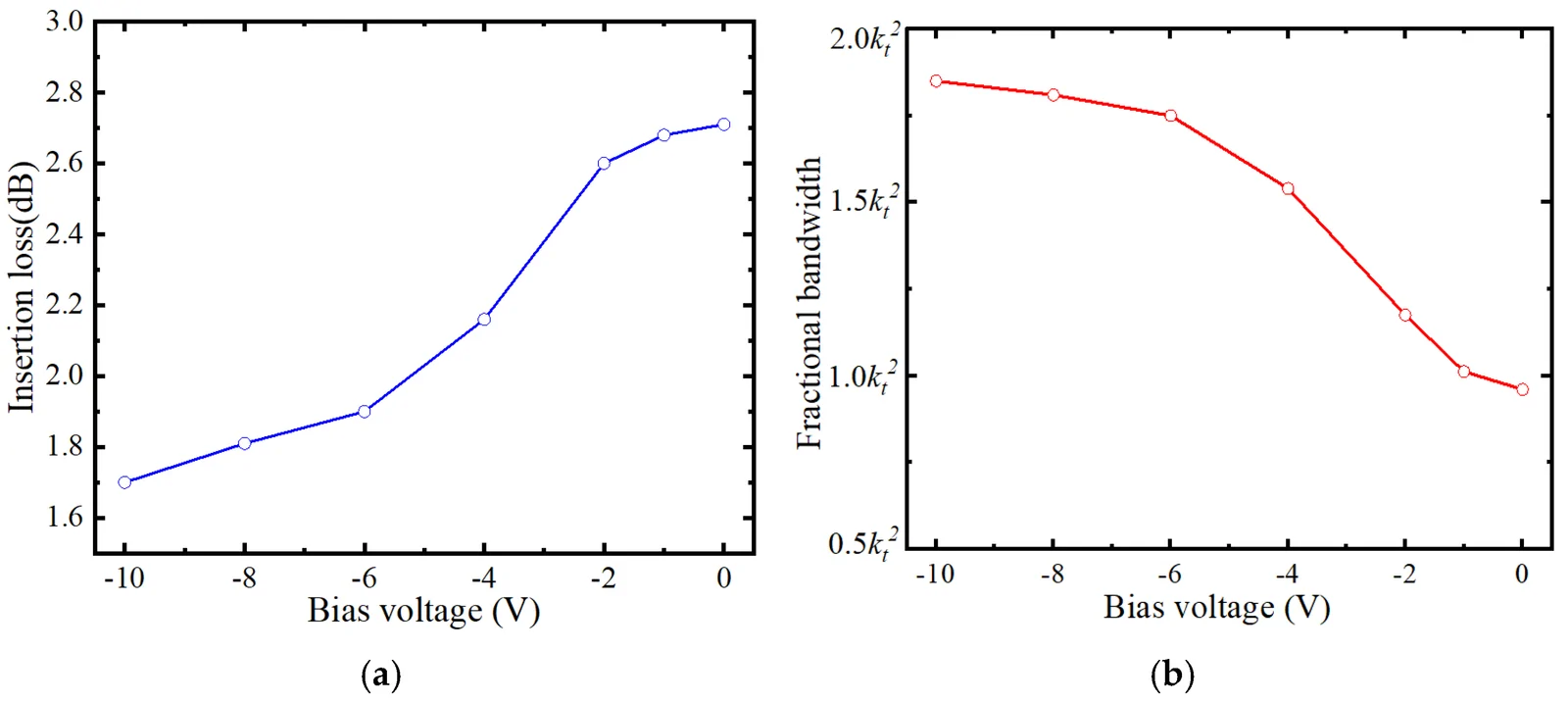
(**a**) Measured insertion loss (Type II); (**b**) measured fractional bandwidth of the reconfigurable hybrid filter (Type II).

Figure 21 presents the measured frequency responses of the Type II reconfigurable filter for four bias conditions: 0 V, −3 V, −5 V, and −10 V. Figure 22a,b present the measured insertion loss and 3 dB fractional bandwidth under different varactor bias voltages, respectively. As the bias voltage increases from 0 V to −10 V, the insertion loss varies from 1.7 dB to 2.7 dB, and the 3 dB fractional bandwidth increases from 0.96*k_t_*^2^ to 1.85*k_t_*^2^, which corresponds to an absolute bandwidth increase from 0.77 MHz to 1.48 MHz, representing a 93% enhancement. In addition, the Type II filter exhibits a better out-of-band rejection, which is approximately 10 dB higher than that of the Type I filter. This is attributed to the additional reconfigurable shunt AWLR-module branch, which introduces a new transmission zero to improve the stopband. It also causes larger insertion loss. These experimental results are in good agreement with the theoretical predictions. The results further validate the proposed design methodology.

### 3.3. Comparison with State-of-the-Art AWLR-Based Filters

A comparison between the proposed Type II filter and previously reported AWLR-based reconfigurable filters is summarized in Table 3. References [26,27] reported reconfigurable AWLR-based filters with bandpass-to-bandstop and all-pass-to-bandstop transfer functions, respectively. Reference [28] presented a three-channel switchable hybrid filter. Reference [29] demonstrated a hybrid bandstop filter with tunable stopband bandwidth. However, none of these works study the continuous bandwidth tunability in bandpass mode. References [30,31] reported AWLR-based filters with tunable passband bandwidth. Their circuits consist of lumped-element resonators and impedance inverters, requiring approximately 35 to 50 components. Their insertion losses range from 1.55 dB to 6.9 dB. In this paper, the proposed reconfigurable hybrid filter employs fewer components while achieving comparable bandwidth tunability and maintaining low insertion loss.

## 4. Conclusions

In this paper, a low-loss hybrid acoustic filter with continuously tunable fractional bandwidth has been proposed. The filter based on a ladder-type configuration employs varactor diodes to achieve continuous bandwidth tuning. Two configurations, named Type I and Type II, are designed. A theoretical analysis based on the two designed configurations is presented. The calculated insertion loss and 3 dB fractional bandwidth show good consistency with those of the simulation. With this method, the electrical characteristics of the reconfigurable hybrid filter can be predicted. The two configurations are fabricated and measured. The Type I filter achieves a fractional bandwidth tuning range from 1.16*k_t_*^2^ to 1.76*k_t_*^2^ with a minimum insertion loss of 1.2 dB and a maximum of 2.2 dB. The Type II filter provides a wider tuning range from 0.96*k_t_*^2^ to 1.85*k_t_*^2^ with a minimum insertion loss of 1.7 dB and a maximum of 2.7 dB. It demonstrates a favorable trade-off between tunability and insertion loss.

## Figures and Tables

**Table 1:** BVD model parameters of the TC0528A SAW resonator.

Symbol	Quantity	Values
*L_m_*	Motional inductance	15.265 μH
*C_m_*	Motional inductance	1.659 fF
*R_m_*	Motional resistance	12.8 Ω
*C*_0_	Static capacitance	1.93 pF

**Table 2:** Lumped-element parameters of the reconfigurable hybrid filter.

Symbol	Quantity	Values
*L_s_*_1_	Shunt inductor in the series branch	12 nH
*L_s_*_2_	Series inductor in the series branch	1 nH
*L_p_*_1_	Series inductor in the shunt branch	16 nH

**Table 3:** Performance comparison with state-of-the-art AWLR-based reconfigurable filters.

Ref.	Reconfigurable Modes	FBW^3^ Tuning Range (*xk_t_*^2^)	IL^4^ (dB)	Component Count
[26]	Bandpass-to-bandstop	N/A (mode switching)	1.5	~16
[27]	All-pass-to-bandstop	N/A (mode switching)	0.38	~25
[28]	Single-to-multi BPF^1^	N/A (mode switching)	2.37–4.25	~55
[29]	Bandwidth-tunable BSF^2^	0.89*k_t_*^2^–2.62*k_t_*^2^	1.7	~20
[30]	Bandwidth-tunable BPF	0.57*k_t_*^2^–1.32*k_t_*^2^	2.2–3.9	~40
[6]	Bandwidth-tunable BPF	0.36*k_t_*^2^–1.35*k_t_*^2^	3.3–6.9	~50
[31]	Tunable bandpass-to-bandstop	0.37*k_t_*^2^–1.33*k_t_*^2^	1.55–5.8	~35
This work	Bandwidth-tunable BPF	0.96*k_t_*^2^–1.85*k_t_*^2^	1.7–2.7	15

BPF^1^: bandpass filter, BSF^2^: bandstop filter, FBW^3^: fractional bandwidth, IL^4^: insertion loss.

## Data Availability

The research data will be provided upon approval by the author.
